# LCD-Composer: an intuitive, composition-centric method enabling the identification and detailed functional mapping of low-complexity domains

**DOI:** 10.1093/nargab/lqab048

**Published:** 2021-05-26

**Authors:** Sean M Cascarina, David C King, Erin Osborne Nishimura, Eric D Ross

**Affiliations:** Department of Biochemistry and Molecular Biology, Colorado State University, Fort Collins, CO 80523, USA; Department of Biochemistry and Molecular Biology, Colorado State University, Fort Collins, CO 80523, USA; Department of Biochemistry and Molecular Biology, Colorado State University, Fort Collins, CO 80523, USA; Department of Biochemistry and Molecular Biology, Colorado State University, Fort Collins, CO 80523, USA

## Abstract

Low complexity domains (LCDs) in proteins are regions predominantly composed of a small subset of the possible amino acids. LCDs are involved in a variety of normal and pathological processes across all domains of life. Existing methods define LCDs using information-theoretical complexity thresholds, sequence alignment with repetitive regions, or statistical overrepresentation of amino acids relative to whole-proteome frequencies. While these methods have proven valuable, they are all indirectly quantifying amino acid composition, which is the fundamental and biologically-relevant feature related to protein sequence complexity. Here, we present a new computational tool, LCD-Composer, that directly identifies LCDs based on amino acid composition and linear amino acid dispersion. Using LCD-Composer's default parameters, we identified simple LCDs across all organisms available through UniProt and provide the resulting data in an accessible form as a resource. Furthermore, we describe large-scale differences between organisms from different domains of life and explore organisms with extreme LCD content for different LCD classes. Finally, we illustrate the versatility and specificity achievable with LCD-Composer by identifying diverse classes of LCDs using both simple and multifaceted composition criteria. We demonstrate that the ability to dissect LCDs based on these multifaceted criteria enhances the functional mapping and classification of LCDs.

## INTRODUCTION

Protein sequence complexity is a measure of the diversity of amino acids found in a sequence. Proteins lie along a finite spectrum of sequence complexity constrained by protein length and the amino acid ‘alphabet’ (generally, the 20 canonical amino acids). While the majority of protein sequences are composed of a diverse mixture of the possible amino acids, a substantial number of proteins contain low-complexity domains (LCDs) composed of only a small subset of the possible amino acid residues. Proteins with LCDs participate in a wide array of molecular processes and have been associated with unique structural, functional and regulatory tendencies (1–24). Additionally, a variety of human diseases are associated with mutation or expansion of LCDs (6,13,25,26).

A variety of methods have been developed to distinguish LCDs from regions of moderate or high sequence complexity, including SEG (27), CAST (28), fLPS (29) and others (30–33), and many of these methods were recently combined in a meta-server for LCD identification (34). However, these methods rely on mathematical definitions of sequence complexity or statistical enrichment of amino acids (relative to whole-proteome frequencies) to distinguish LCDs from complex sequences. Although these methods provide well-defined cutoffs for LCDs, they do not intuitively correspond to biochemical features, making it difficult for researchers to customize search parameters for desired purposes. Additionally, LCDs can be further decomposed into classes based on which specific amino acid(s) are most common. While the amino acids are often treated equivalently by sequence complexity methods, the actual physical properties of the amino acids can be radically different, resulting in LCDs with completely distinct physical behavior.

For example, although the SEG algorithm has been used effectively to identify LCDs for biochemical characterization, its original intended purpose was for the masking of LCDs to improve sequence alignment, and it is still used in the pervasive BLAST tool (35). Consequently, SEG does not distinguish between LCDs of different classes (e.g. N-rich LCDs versus K-rich LCDs). A search for protein domains with a given complexity score will return a highly heterogeneous mixture of LCDs with dramatically different compositions (and therefore different structural and functional behaviors), requiring additional downstream sequence analysis to narrow results to specific LCDs of interest with particular compositional features.

Likewise, although methods that use statistical overrepresentation of specific amino acids have numerous applications, they face a different set of limitations. Specifically, while a protein's amino acid composition is directly linked to its physical properties, amino acid overrepresentation is only indirectly linked. Although it is possible in some cases for users to vary the parameters for statistical overrepresentation methods to identify thresholds that mimic composition-based approaches (though only for the most basic LCD searches), a method that directly detects amino acid composition is likely to be simpler and more intuitive for researchers interested in searching for domains that meet specific compositional thresholds. Furthermore, the simplicity of a composition-based approach enables intuitive, multifaceted searches for LCDs enriched in multiple amino acids or groups of amino acids at different composition thresholds, which are not currently built-in features of existing methods employing statistical enrichment.

Finally, since protein sequence complexity exists along a spectrum, a single complexity threshold, though often useful, may not always be biologically relevant (1). Consequently, different complexity thresholds may be suitable depending on the types of LCDs of interest and the research question at hand. However, with both approaches, choosing a threshold for sequence complexity or statistical overrepresentation for a specific LCD search purpose will often require extensive experimentation, optimization, or prior calculations, since neither a complexity score (such as <2.2 bits) or a statistical overrepresentation (such as *P* < 10^–3^) is intuitively linked to a protein's physical properties.

Here, we report a new computational tool, the low-complexity domain composition scanner (LCD-Composer), which defines LCDs in proteins based on amino acid composition and linear dispersion of amino acids. The primary intended purpose of LCD-Composer is the intuitive identification of LCDs with a focus on the predominant physicochemical characteristics of the LCDs. LCD-Composer is a stand-alone Python script (requiring no external packages, downloads, or configuration) that runs on all operating systems. The algorithm completes full-proteome scans in seconds, and runtime scales linearly with proteome size, permitting whole-proteome or multi-proteome analyses. Optional LCD-Composer parameters are customizable, allowing for both simple and multifaceted compositional constraints that can be specified by users. Together, these features make LCD-Composer intuitive, accessible to researchers with limited computational experience, and suitable for diverse research applications. Additionally, we demonstrate the unique ability of LCD-Composer to rapidly identify both simple and multifaceted LCDs with high specificity, and to dissect LCDs into distinct subclasses of functional importance across an array of model organisms.

## MATERIALS AND METHODS

### Calculation of amino acid composition and linear amino acid dispersion

LCD-Composer implements a sliding window approach (with a 20aa default window size, and a step size = 1) to evaluate local amino acid composition. For each window, the amino acid composition, *C*, is calculated as the sum of the total occurrences of each amino acid in the specified set divided by the length of the sequence:

$$\begin{equation*}C = \frac{{\mathop \sum \nolimits_{r \in A} \;{n_r}}}{L}\;\end{equation*}$$

where *A* represents the set of specified amino acids, *n_r_* represents the number of times residue *r* occurs in the window sequence, and *L* represents the window size used (or the length of the sequence being analyzed).

Let *B* represent the set of the canonical amino acids not in set *A*. The linear dispersion of residues in the chosen set *vis-à-vis* all other residues and the sequence termini is calculated as the normalized standard deviation of the spacing of residues in set *A* and the spacing of residues in set *B*, with sequence termini included in the consideration. Specifically, for a given protein sequence, the differences in numerical position for all residues in set *A* from the nearest neighbor of the same set and from the sequence termini are calculated. This procedure is repeated for all residues in set *B*. The spacing values are then combined into a single array, and the standard deviation *s* of the array is calculated as:

$$\begin{equation*}s = \sqrt {\frac{{\sum {{\left( {{d_i} - \;\bar d} \right)}^2}}}{N}} \end{equation*}$$

where *d_i_* represents the difference between the position of the *i*th residue and the position of the previous residue from among the corresponding set (or the sequence terminus) in the given protein sequence, $\bar d$ represents the mean of the spacing values, and *N* represents total number of differences calculated. For searches with multiple specified groups of amino acids, residues from all groups are combined into a single set, and their linear dispersion *vis-à-vis* all other residues is calculated. Note that, while this enhances the sensitivity of detecting LCDs with multi-faceted search criteria by mitigating exclusion of domains on the basis of insufficient dispersion of amino acid(s) with low composition thresholds, in rare instances this can identify regions with adjacent LCDs that are not well-mixed.

Since the length and composition of a sequence determines the range of possible values for the standard deviation of linear spacings, the standard deviation *s* is then normalized to the range of possible values:

$$\begin{equation*}{s_{norm}} = 1 - \left( {\frac{{s - {s_{min}}}}{{{s_{max}} - {s_{min}}}}} \right)\end{equation*}$$

where *s_min_* and *s_max_* are standard deviations calculated from two artificially generated sequences of identical length and composition designed to minimize and maximize *s*, respectively. *s_min_* is obtained when the specified amino acid is distributed as uniformly as possible across the sequence window, which occurs when the absolute difference between $\bar d$ and ${d_{i\;}}$is <1 for all spacing values; when multiple sequences fit these criteria, the sequence was chosen in which the larger values for ${d_i}$ are at the N-terminus. *s_max_* is obtained when the specified amino acid is entirely clustered at one end of the sequence. This method of determining *s_min_* and *s_max_* was validated on exhaustive sets of sequences ranging from 5aa to 30aa in length, and we expect that it should scale to all window sizes (see Supplementary Material). The final linear dispersion *s_norm_* is on a scale from 0 to 1, where larger values indicate increased linear dispersion of the amino acid(s) of interest (i.e. well-mixed sequences). By default, LCD-Composer ignores the linear dispersion parameter if the composition of the amino acid(s) of interest exceeds the midpoint between the user-specified composition threshold and 100% in order to correct for sequences of very high composition but containing intervening gaps between residues of interest resulting in a low linear dispersion (see Supplementary Figure S3 in Supplementary Material). However, users can also specify a composition value at which the linear dispersion parameter is ignored using the ‘-i’ flag (e.g. ‘-i 75’ to ignore the linear dispersion parameter for sequences with >75% composition of the amino acid of interest). Additionally, all regions for which 100% of the residues are among the amino acids of interest are automatically identified as an LCD regardless of chosen linear dispersion parameters.

### Merging and trimming of identified domains

After each protein is scored, any overlapping domains that pass the user-specified amino acid composition and linear spacing thresholds are merged into a single domain. All other regions are masked, unless the verbose option is employed, in which case all regions are scored regardless of whether they pass the user-specified thresholds. For each merged domain, both termini are trimmed until the amino acid at each terminus matches an amino acid from the user-defined set of residues. After final processing, the overall composition (with respect to the user-defined set of amino acids) and linear dispersion is calculated for each merged/trimmed domain. In rare cases, merging and trimming of the domain may result in a composition or linear dispersion that is slightly lower than the user-defined threshold—this behavior is intentional and allowed since the identification and merging of underlying windows maintains strict adherence to the user-defined composition and linear dispersion thresholds.

For each protein containing at least one domain of interest, all identified domains, corresponding domain boundaries, final domain compositions, and final normalized standard deviations of linear spacings are written to an output file. Additionally, if the verbose option is implemented, per-position compositions and per-position linear dispersion values (up to the length of the sequence minus the window size) are included in the results.

### Whole-proteome analyses, parameter benchmarking, and speed tests

For in-depth analyses of specific proteomes, the yeast proteome (*Saccharomyces cerevisiae*, UniProt ID UP000002311) was downloaded from the UniProt website on 12/25/2019. Proteomes for model eukaryotic organisms [*Caenorhabditis elegans* (nematode), UP000001940; *Drosophila melanogaster* (fruit fly), UP000000803; *Danio rerio* (zebrafish), UP000000437; *Xenopus laevis* (African clawed frog), UP000186698; *Mus musculus* (mouse), UP000000589; and *Homo sapiens* (human), UP000005640] were initially downloaded from the UniProt website on 11/19/2020 for proteomes with only one protein sequence per gene or 2/23/2020 for proteomes containing all known isoforms. For evaluation of simple LCDs across all organisms on UniProt, all available proteomes for archaea, bacteria, and eukaryote were downloaded from the UniProt FTP server (ftp://ftp.uniprot.org/pub/databases/uniprot/) on 21 August 2020. All virus proteomes were downloaded from the same site on 23 August 2020–24 August 2020. Proteomes UP000011843_306025, UP000202407_908070 and UP000269945_48420 were excluded from further analyses due to unusually small proteome sizes. Protein sequences were parsed using the Biopython (version 1.76) FASTA parsing module (36). All analyses involving speed tests were run on a simple desktop computer [HP EliteDesk 800 G2, with Intel Core i7-6700 processor (3.40GHz) and 8GB RAM] operating on Windows 10. For parameter benchmarking, the yeast proteome was analyzed for each amino acid, window size, linear dispersion threshold, and minimum composition threshold, with a single parameter varied each time and the remaining parameters fixed as the default values (window size = 20aa, linear dispersion threshold = 0.5, minimum composition threshold = 40%). For GO term analyses, the gene ontology file was downloaded from http://geneontology.org/ on 27 February 2020. GO annotation files for all organisms were downloaded from ftp://ftp.ebi.ac.uk/pub/databases/GO/goa/ on 27 February 2020. GO enrichment analyses were performed using the GOATOOLS (version 1.0.2) library, with the propagate_counts option set to ‘False’ to reduce the proportion of broad/non-specific GO terms among statistically significant results (37).

### Statistical estimation of cross-organism GO term counts and secondary amino acid enrichment within LCDs

Certain GO terms are statistically associated with long proteins, which can increase the type I error rate using standard GO methodology despite multiple hypothesis test correction. To account for this, cross-organism GO term enrichment counts were estimated by length-weighted random sampling of proteins from each proteome and evaluation of the number of times the same GO term was observed in multiple organisms. For each LCD class and organism, proteins were randomly sampled (without replacement, weighted proportionally by protein length) until the sample size matched that of the observed sample size for the same LCD class and organism, then evaluated for enriched terms by GO analysis. For each GO term identified in any of the organisms, the number of times it occurred across the seven organisms was calculated. This procedure was repeated 1000 times for every combination of LCD class and organism, resulting in ∼140k total GO term tests and ∼20k cross-organism tests. Note that the probability of an LCD occurring in a protein may not scale linearly with protein length when more than one LCD is likely to occur in a protein of given length: in such cases, our method of estimating the effect of protein length on type I error rate likely results in conservative estimates of GO term enrichment counts (i.e. inflated numbers of enriched GO terms derived from sampling).

To estimate the number of times identical GO terms would be sampled in multiple organisms assuming the enriched GO term sample size for the LCD-containing protein sets, GO terms were iteratively sampled for each organism. Specifically, for each LCD class, GO terms were randomly selected (without replacement) for each organism from a complete set of GO terms containing at least one directly annotated gene product in that organism until the sample size matched the observed number of enriched GO terms. The number of times each sampled GO term occurred across the sampled lists was then calculated and stored. This procedure was repeated for a total of 100k iterations. Observed cross-organism GO term counts were then statistically compared to the cross-organism GO term counts derived from iterative sampling using a two-sided Fisher's exact test, with Bonferroni correction for multiple hypothesis testing applied within each LCD class (seven possible cross-organism count categories for each LCD class).

Secondary amino acid enrichment was calculated by first exhaustively scanning the yeast proteome with a 20aa window size for each amino acid. For each of the 19 remaining secondary amino acids, the number of windows for which that amino acid was either (i) unambiguously the most abundant, or (ii) the second-most abundant behind only the primary amino acid, was tallied. The degree of enrichment or depletion (*E*) for each LCD subclass (*s*) was calculated as:

$$\begin{equation*}{E_s} = {\rm{ln}}\left( {O{R_s}} \right)\end{equation*}$$

and

$$\begin{equation*}O{R_s} = \;{{\left( {\frac{{{f_{{s_{obs}}}}}}{{1 - {f_{{s_{obs}}}}}}} \right)} \mathord{\left/ {\vphantom {{\left( {\frac{{{f_{{s_{obs}}}}}}{{1 - {f_{{s_{obs}}}}}}} \right)} {\left( {\frac{{{f_{{s_{wp}}}}}}{{1 - {f_{{s_{wp}}}}}}} \right)}}} \right. } {\left( {\frac{{{f_{{s_{wp}}}}}}{{1 - {f_{{s_{wp}}}}}}} \right)}}\;\end{equation*}$$

where *f_s_obs__* represents the fraction of the total observed primary LCDs assigned to the given LCD subclass (*s*), and *f_s_wp__* represents the fraction of windows encountered during the whole-proteome scan for which the secondary amino acid was most abundant (again, excluding the primary amino acid). Subclasses for which the scaled whole-proteome frequency (i.e. the fraction of windows assigned to the LCD subclass multiplied by the total observed primary LCDs) was <1 were excluded from analyses. Subclasses for which the scaled whole-proteome frequency ≥1 but with no observed LCDs assigned to that subclass were assigned an imputed value of 1 for the observed LCD frequency to provide a conservatively biased estimate. *P*-values were calculated using a two-sided Fisher's exact test, with Bonferroni correction for multiple hypothesis testing.

## RESULTS

### LCD-Composer: identification and demarcation of LCDs

Compared with sequence complexity or statistical amino acid bias, amino acid composition more closely reflects the physicochemical properties of LCDs in proteins. Additionally, a direct readout of amino acid composition is likely to be more intuitive to cellular and molecular biologists than a statistical score of complexity or bias. However, one limitation of using amino acid composition alone to define LCD boundaries is the occurrence of LCDs which pass the specified composition criteria (e.g. 50% Q, for Q-rich domains) but exhibit an asymmetric distribution of the amino acid of interest. For example, Q residues constitute 50% of the sequence QQQQQPGTRR, but the residues at the C-terminus are unrelated to the LCD of interest. The spacing of particular amino acids is an important determinant of biophysical behavior across a variety of LCDs (38–45). Therefore, we considered a second parameter, the distribution of the amino acid(s) of interest across the sequence, as an important feature capable of further resolving LCDs of similar or identical compositions.

**Figure 1.**
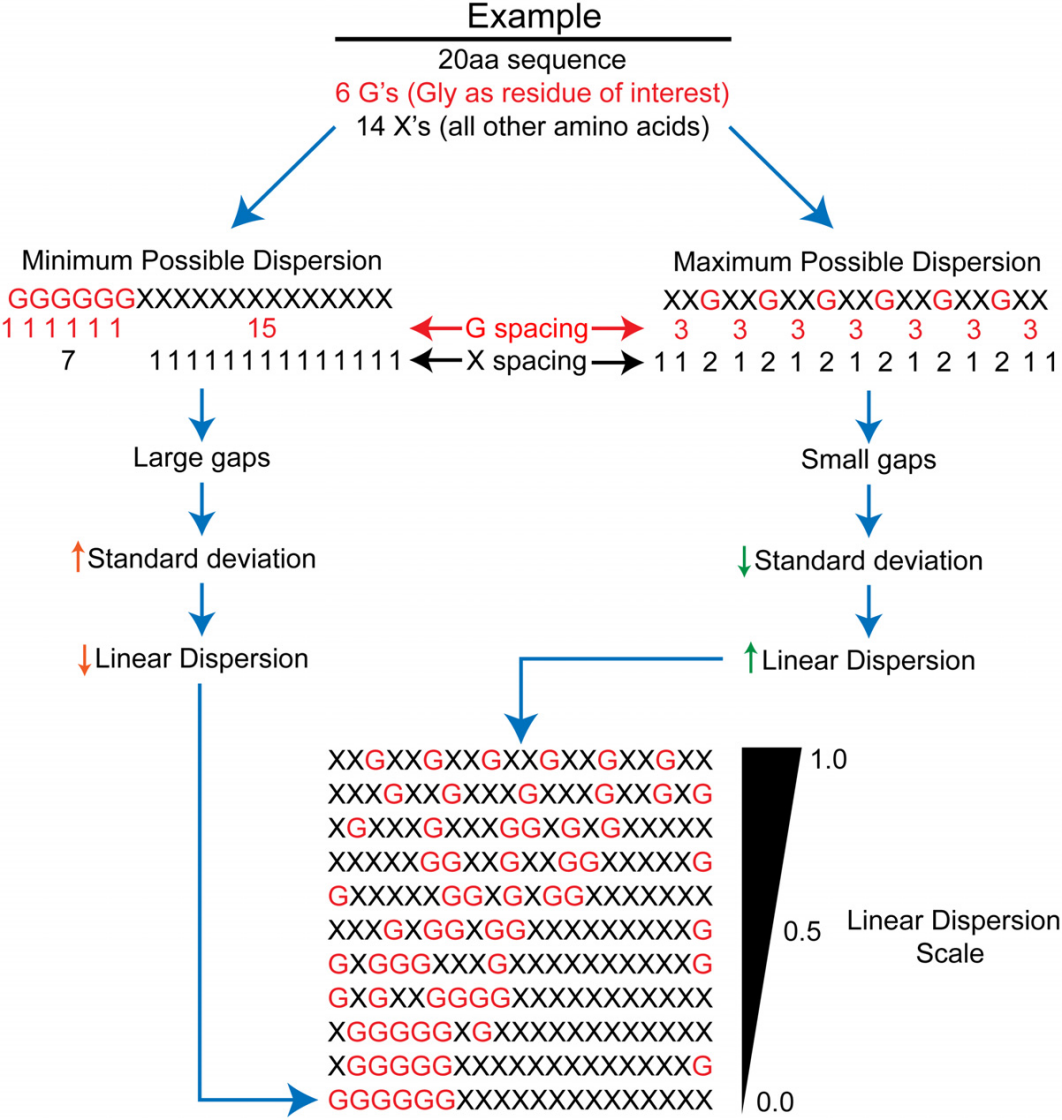
Depiction of linear dispersion parameter. Linear dispersion is calculated from the normalized standard deviation in the combined spacing values for all residues of interest and all other residues. Large gaps lead to large standard deviations, resulting in low linear dispersion values. Conversely, small gaps with uniform spacing leads to small standard deviations and high linear dispersion values. The linear dispersion scale ranges from 0.0 to 1.0, with high linear dispersion values indicating well-mixed sequences.

To measure the spacing of amino acids in protein sequences, we derived a basic procedure to quantify the normalized standard deviation of the spacing of a specified amino acid (or set of amino acids) relative to each other and relative to the termini of a given window sequence (Figure 1; see Material and Methods and Supplementary Material for detailed descriptions). This statistic, which we refer to as the ‘linear dispersion’ of amino acids, was tested on an exhaustive series of benchmark sequences consisting of all possible 20-residue sequences composed of two representative amino acids (see Supplementary Figures S1–S3 for extensive analysis and discussion of the linear dispersion parameter).

**Figure 2.**
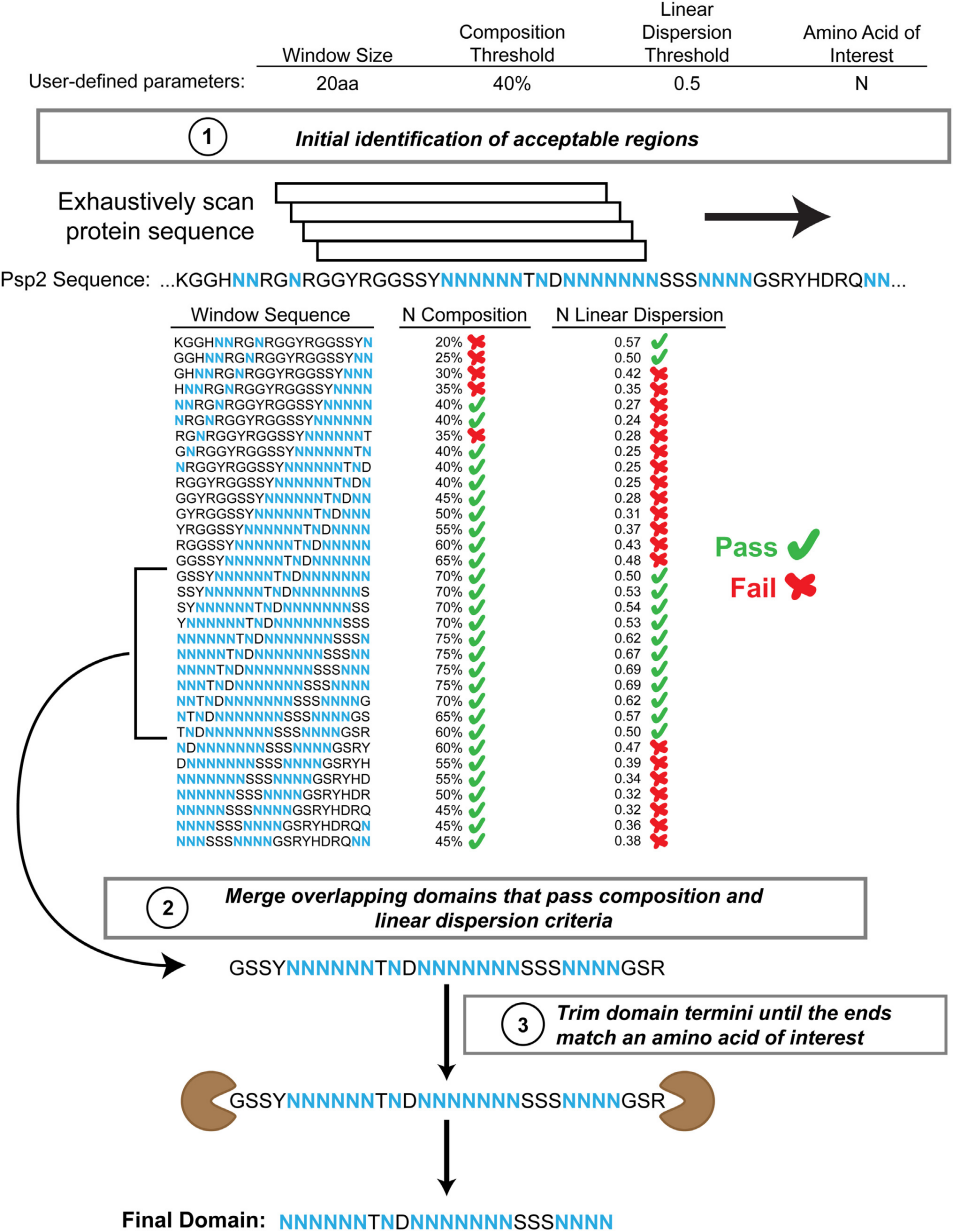
Computational procedure for identifying LCDs of interest. Identification of LCDs occurs in two stages. (**A**) In the first stage, protein sequences are scanned using a sliding window. For each window subsequence, the percent composition of the amino acid(s) of interest and its linear dispersion are calculated. (**B**) In the second stage, overlapping domains that pass the composition and linear dispersion criteria are merged into a single domain, then trimmed such that the final residue at both termini are an amino acid of interest.

These two parameters—amino acid composition and linear dispersion of amino acids—were combined into a single computational approach to identify and demarcate LCDs (Figure 2). This method, which we call LCD-Composer, is available as a stand-alone command-line script written in Python (https://github.com/RossLabCSU/LCD-Composer). Briefly, LCD-Composer uses a sliding window to scan protein sequences. For each subsequence, the percent composition and linear dispersion corresponding to the amino acid (or group of amino acids) of interest are calculated. Overlapping subsequences that pass the user-specified composition and linear dispersion criteria are merged into a single domain. Domain termini are then trimmed until an amino acid of interest is the ultimate residue at both extremes of the domain, resulting in the final LCD.

LCD-Composer offers a variety of optional parameters that can be specified by users at runtime to tailor LCD-Composer behavior to suit individual purposes. Optional parameters include scanning window size (default = 20aa), minimum percent composition threshold (default = 40), minimum linear dispersion threshold (default = 0.5), and an amino acid or group of amino acids of interest. To help guide the choice of non-default parameters, the effects of varying each parameter on LCD identification and definition were systematically evaluated and are included in Supplementary Figures S4 and S5. Additionally, we evaluated the speed and scalability of LCD-Composer on a variety of model proteomes. LCD-Composer is reasonably fast (∼4 s and ∼30 s for analysis of the yeast and human proteomes, respectively, on a basic desktop computer; see Material and Methods) with a computation time that scales linearly with proteome size (Supplementary Figure S6), making it suitable for multi-proteome analyses.

**Figure 3.**
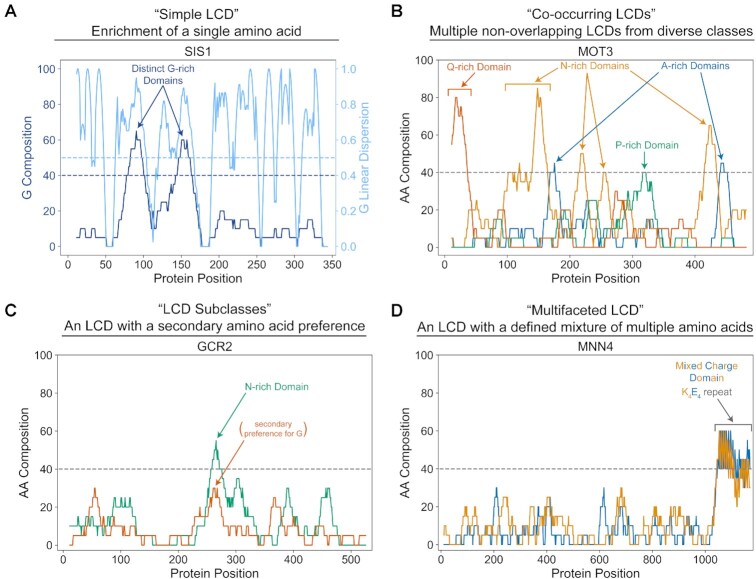
Examples of LCD contexts within individual proteins. (**A**) An LCD-Composer scan of the Sis1 protein identifies two distinct G-rich domains that pass the composition and linear dispersion thresholds. (**B**) A complete LCD-Composer scan searching for all possible types of single-amino acid LCDs identifies multiple non-overlapping LCDs of distinct classes in the Mot3 protein. (**C**) The Gcr2 protein contains an N-rich domain with a subsidiary preference for G. (**D**) The Mnn4 protein contains a multifaceted LCD with a high and roughly balanced K/E composition.

To highlight the diversity of LCD features and contexts, we identified proteins with specific types of LCDs or combinations of LCDs (Figure 3). We broadly classify these situations into four main categories: (i) proteins with only a single type of LCD (‘simple LCDs’; Figure 3A); (ii) proteins with multiple, non-overlapping LCDs from distinct classes (‘co-occurring LCDs’; Figure 3B); (iii) LCDs that exhibit a clearly predominant amino acid, but also exhibit a subsidiary preference for a second type of amino acid (‘LCD subclasses’; Figure 3C) and (iv) LCDs that can be characteristically defined by enrichment of multiple types of amino acids (‘multifaceted LCDs’; Figure 3D). Each of these situations is evaluated in greater detail below.

### A comprehensive survey of simple LCDs and organisms with extreme LCD content across all domains of life

The computational efficiency of LCD-Composer is sufficient to perform high-throughput analyses on multiple proteomes in a relatively short span of time. To gain a broad perspective of whole-proteome LCD content within and across domains of life (we refer to viruses as a ‘domain of life’ for simplicity only), we ran LCD-Composer for each amino acid using default parameters on all reference proteomes available on the UniProt website (*n* = 18 896). All identified LCDs are available as a supplementary resource at (46).

**Figure 4.**
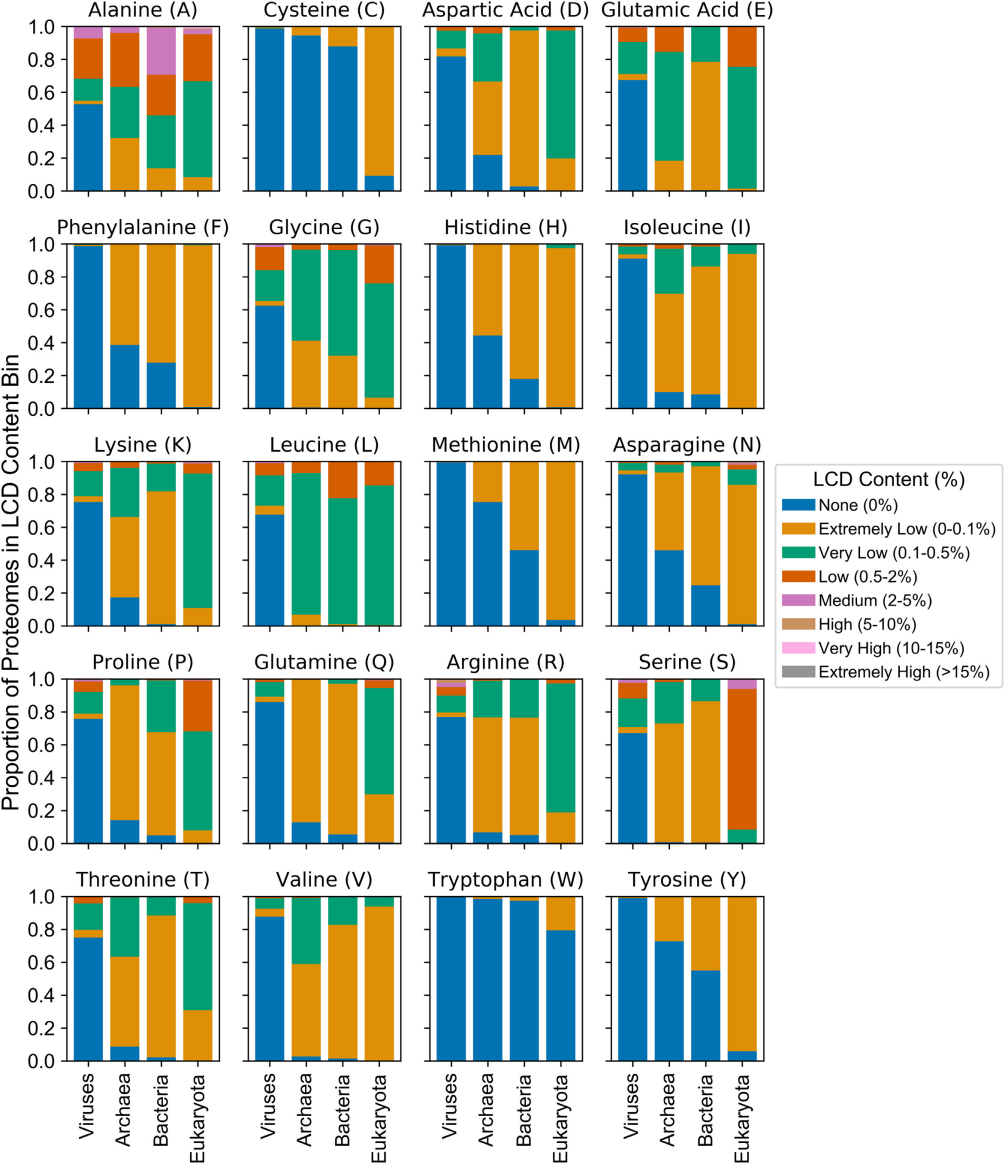
Cross-domain comparison of LCD content across all proteomes for each LCD class. LCDs were identified using LCD-Composer with default parameters for all proteomes available from UniProt. For each LCD class, the percentage of each proteome classified as LCD was defined as the percentage of amino acids lying within LCD regions out of the total proteome size (in number of amino acids). Within each domain of life, organisms were then sorted into one of seven categorical bins based on the percentage of the proteome classified as LCD for each LCD class [none (0%), extremely low (0–0.1%), very low (0.1–0.5%), low (0.5–2%), medium (2–5%), high (5–10%), very high (10–15%), or extremely high (>15%)]. The proportion of total proteomes for each domain of life was then calculated for each bin and plotted as a stacked bar chart. For all organisms, the ‘additional’ file containing sequences of known protein isoforms (when available) was combined with the corresponding organism's main proteome prior to analyses.

To explore gross differences in whole-proteome LCD content between domains of life, the percentage of each proteome classified as LCD was calculated for each LCD class. Proteomes were then binned within each domain of life based on the percentage of the proteome classified as LCD for each LCD class (Figure 4 and Supplementary Tables S1, S2). For most amino acids, the proportion of organisms with at least some LCD content progressively increases in the order viruses→archaea→bacteria→eukaryota. However, the different domains of life showed distinct biases in terms of which class of LCDs was most likely to be highly enriched. For example, S-rich LCDs constitute >0.5% of each proteome for nearly all eukaryotic organisms, yet S-rich LCD content rarely exceeds 0.1% for the majority of archaeal, bacterial, and viral organisms. By contrast, bacteria were far more likely than other types of organisms to have a relatively high (>2%) A-rich LCD content.

**Figure 5.**
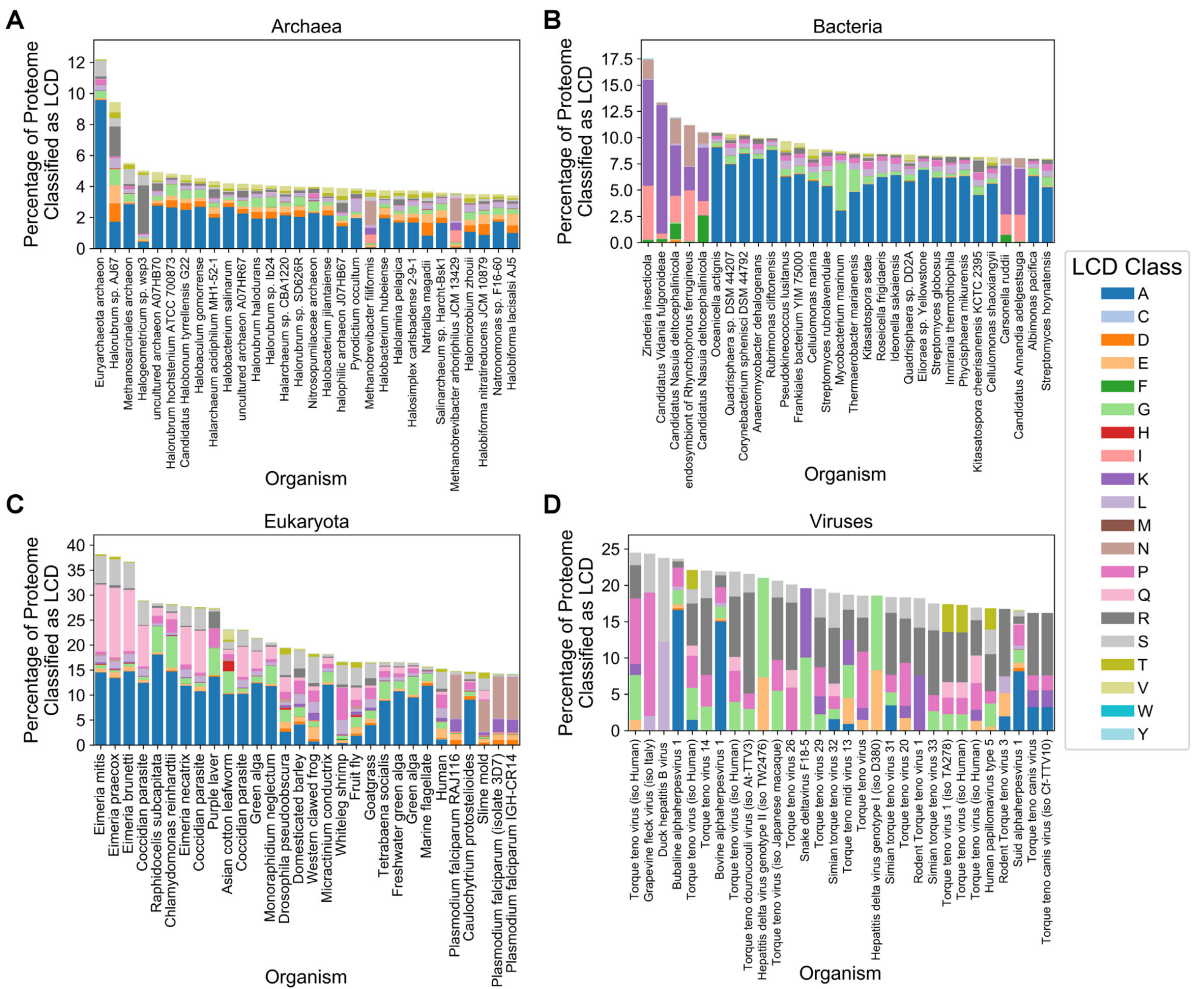
Cross-domain comparison of organisms with the highest total LCD content. For each domain of life, the total percentage of LCD content was calculated as the sum of the individual LCD content percentages for each LCD class (note that, in some cases, this method results in a slight overestimation of total LCD content due to overlapping LCDs from different classes but was chosen to preserve LCD percentages for individual LCD classes). Organisms were then ranked from highest to lowest and the LCD percentages (parsed by LCD class) were plotted for the top 30 organisms for Archaea (**A**), Bacteria (**B**), Eukaryota (**C**) and Viruses (**D**). LCD percentages for all organisms (including those ranking below the top 30) and all LCD classes are available in Supplementary Table S1.

While the majority of organisms contain relatively low LCD content for each LCD class, we were intrigued by the small proportion of organisms that contain an unusually high percentage of their proteome classified as LCD. To explore organisms from each domain with the highest overall LCD content, the total LCD content was determined for each organism by summing the percentage of the proteome classified as LCD across all LCD classes. Eukaryotic organisms achieve the most extreme overall LCD content (∼15–38% for the top 30 organisms), followed by viruses, bacteria, and archaea, respectively (Figure 5). The LCD content profiles for high-LCD organisms differs substantially between domains of life. For example, high-LCD archaea tend to have higher proportions of negatively charged (D- or E-rich), T-rich, and V-rich LCDs compared to high-LCD organisms from other domains (Figure 5A). The top 5 bacterial organisms contain unusually high proportions of I-rich, K-rich, and N-rich LCDs, whereas the majority of the remaining 25 organisms tend to have an extremely high percentage classified as A-rich LCD (Figure 5B). High-LCD eukaryotic organisms tend to have a high percentage of A-rich and S-rich LCD coupled with either a high proportion of Q-rich LCD or G-rich LCD. Interestingly, humans are among the top 30 organisms (out of 1473) in terms of total LCD content, yet exhibit a remarkably diverse LCD profile consisting predominantly of A-, E-, G-, K-, L-, P-, Q-, R-, S- and T-rich LCDs (Figure 5C). Finally, the majority of high-LCD viruses are torque teno viruses that tend to have high percentages of R-, P-, G- and S-rich LCDs, whereas alphaherpes viruses have high A-, G- and P-rich LCD percentages, and hepatitis viruses exhibit high E- and G-rich LCD percentages (Figure 5D).

Overall these data reveal large-scale trends in LCD content across organisms, identify organisms with extreme LCD content, and serve as an accessible resource for LCDs in all reference proteomes currently available from UniProt. In the ensuing sections, we utilize a limited set of model organisms to explore relationships between LCD composition and LCD function in greater depth.

### Common and unique functions of LCDs across eukaryotic model organisms

As demonstrated in Figures 4 and 5, and consistent with previous research, proteome compositions and the number of instances of each type of LCD often differ between organisms (1,2,5,9,20). However, similar LCDs may perform related functions across organisms owing to shared biophysical properties. To explore common and unique functional relationships for each LCD class across a limited set of model organisms, we collected all LCDs identified within the proteomes of 7 common eukaryotic model organisms (*S. cerevisiae*, *C. elegans*, *D. melanogaster*, *D. rerio*, *X. laevis*, *M. musculus* and *H. sapiens*) and performed a separate Gene Ontology (GO) analysis for each class of LCDs within each organism. The complete list of LCDs identified for each organism is provided in Supplementary Table S3 (127 472 distinct LCDs across the seven eukaryotic model organisms).

**Figure 6.**
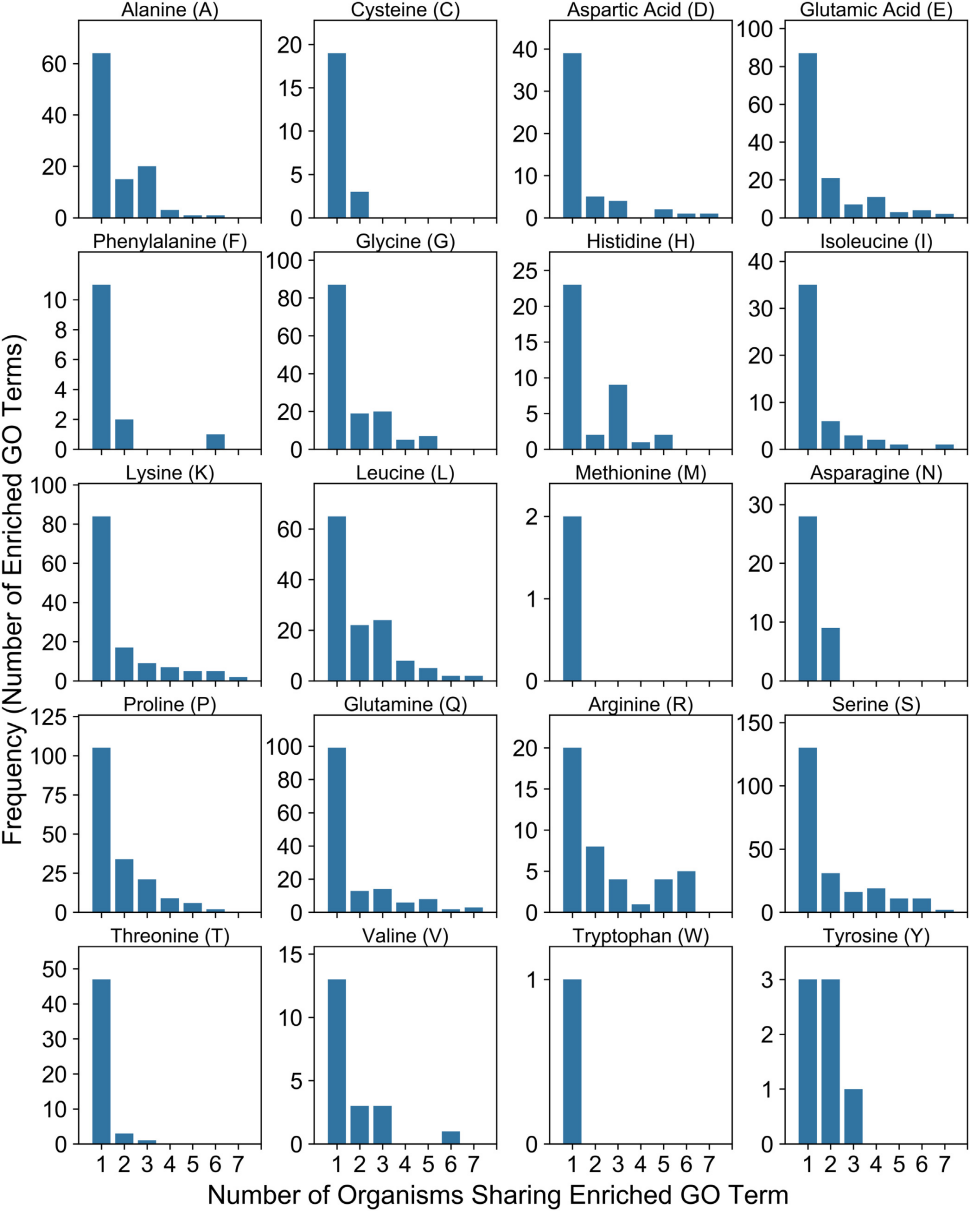
Identification of identical and unique GO terms associated with each LCD class across seven model eukaryotic organisms. GO analyses were performed independently for each LCD class within each eukaryotic model organism. For each LCD class, significantly enriched GO terms were collected for all eukaryotic model organisms in our study. The number of times each enriched GO term occurred across organisms was then calculated and plotted.

For most LCD classes, a substantial number of functional associations are significantly enriched in at least one organism (Figure 6; see Supplementary Table S4 for the number of times each significantly enriched functional annotation was detected across the set of model organisms; complete functional annotation results for all LCD classes for all seven model organisms are provided in Supplementary Table S5). In many instances, an identical GO term was significantly enriched for the same LCD class in more than one organism. Additionally, the mean proportion of overlap in GO terms is unanimously higher for comparisons of the same LCD class across organisms (e.g. A-rich LCDs versus A-rich LCDs) than for comparisons of distinct LCD classes across organisms (e.g. A-rich LCDs versus E-rich LCDs), indicating that the observed GO term conservation is an effect specifically related to each LCD class (Supplementary Figure S7 and Table S6). Finally, similar results are obtained when protein sampling is weighted by protein length (Supplementary Figure S8), all protein isoforms are included in the original LCD analysis (Supplementary Figure S9), GO annotations assigned on the basis of sequence homology are excluded from the gene annotation files (Supplementary Figure S10), or GO terms (rather than proteins) are iteratively sampled (Supplementary Figure S11 and Table S7).

For the majority of LCD classes, ∼15–20% of all enriched GO terms are shared across three or more organisms (Supplementary Figure S12), suggesting that some classes of LCDs are specifically suited for certain cellular and molecular functions across eukaryotes. 175 GO terms spanning 14 LCD classes (A, D, E, F, G, H, I, K, L, P, Q, R, S and V) are significantly enriched for four or more distinct organisms (∼10% of all enriched GO terms). For example, D-rich, E-rich and K-rich LCDs are individually significantly associated with the nucleus and/or nucleolus in all 7 eukaryotic organisms examined (and related functions such as histone, chromatin, and/or DNA binding in six of seven organisms), consistent with previous observations and the known association of highly charged domains with the nucleus/nucleolus (44,47–49). L-rich LCDs are significantly associated with integral membrane proteins involved in transmembrane transport in all seven eukaryotes. Q-rich LCDs are associated with the regulation of transcription by RNA polymerase II in all seven eukaryotes, consistent with previous observations (8,9,19,50). R-rich LCDs are specifically associated with RNA-binding and the regulation of RNA-splicing in six of the seven organisms. S-rich LCDs are associated with an identical set of nine functional annotations related to nuclear localization, DNA-binding, and transcription across six of the seven eukaryotic organisms. While previous studies have uncovered a small subset of these associations (3,6,8,9,19), the composition-centric method employed by LCD-Composer yields, to our knowledge, the most comprehensive set of linkages between LCD properties and their common functions across eukaryotes.

### Multifaceted composition criteria aid in the identification of specific subclasses of LCDs

Some classes of LCDs are characteristically enriched in multiple amino acids, either individually (a single residue from the group comprising the majority of the LCD; Figure 3C) or in combination (co-occurring within the same LCD; Figure 3D). For instance, prototypical yeast prion domains are strongly enriched in Q and/or N residues, but often have a subsidiary bias for Y (19,51), which is important for prion formation (52,53). R/G/Y-rich domains have been associated with liquid-liquid phase separation (LLPS) or liquid–solid gelation, which appear to be related to dynamic interactions in membraneless organelles and/or nuclear pore complexes (44,54–61). Furthermore, the spacing of aromatic residues in certain LLPS-competent domains tends to promote LLPS (38), and R/G/Y composition criteria have already been incorporated into a prediction method for identifying similar domains (62). A P-rich LCD (with additional biases for Q/N/G) modulates the ability of the yeast polyA-binding protein, Pab1, to phase separate in response to stress, and this effect could be tuned by altering the hydrophobicity of the LCD (63). Highly-charged domains often adopt a variety of disordered conformations (41,42), though some highly-charged domains with roughly balanced positive and negative amino acid compositions and regular spacing can form α-helices (39,40). Therefore, in addition to simple single-amino acid searches, LCD-Composer allows for specification of multifaceted composition criteria involving multiple amino acids at different minimum composition percentages.

To illustrate the use of multifaceted composition criteria to identify specific types of LCDs, we ran LCD-Composer on the yeast proteome using composition criteria corresponding to defined features of experimentally characterized LCDs (Table 1). Specification of multifaceted composition criteria primarily works using ‘and’ logic. For example, the command-line option ‘-a QN_Y -c 40_10’ (-a referring to amino acids of interest, and -c referring to corresponding minimum composition thresholds) stipulates that a domain must have a combined Q/N composition exceeding 40% and a Y content exceeding 10%. The combination of these constraints would aid in the identification of domains that are predominantly Q/N-rich but may have a secondary bias for Y. The command-line option ‘-a G_RY -c 30_15’ identifies domains with a primary G enrichment ≥30% and a secondary enrichment of R/Y residues ≥15%. A simple composition analysis of the Pab1 P-rich LCD examined in (63) revealed Q/N, P and G compositions of ∼20%, 19% and ∼15% respectively, with aliphatic residues being important subsidiary components but variable with respect to predominant aliphatic residue across organisms. Conservative composition thresholds based on these values identifies a number of candidate domains that may have related physicochemical behavior. Finally, the composition criteria ‘-a DE_KR -c 40_40’ identifies highly charged domains containing a high fraction of both positively-charged and negatively-charged amino acids. A number of the identified domains exhibit a charge composition and patterning characteristic of charged single α-helices [e.g. Mnn4 and Fpr3; (39,40)], while others have sufficient charge composition but irregular charge spacing (e.g. Pxr1). Therefore, multifaceted composition criteria can (i) result in identification of LCDs whose collective composition exceeds the minimum composition threshold even though the individual amino acid compositions do not, (ii) identify domains with both primary and secondary amino acid biases and (iii) selectively exclude LCDs that would be identified by single-amino acid searches but are not of interest to the user. Importantly, although some LCD-identification methods can identify primary and secondary amino acid biases, they cannot (to our knowledge) simply and specifically search for such domains using separate composition thresholds or customized amino acid groupings.

**Table 1.** Examples of LCDs identified by LCD-Composer with multifaceted composition search criteria. The yeast proteome was evaluated using LCD-Composer with varying search parameters (‘-a’, amino acids used in search; ‘-c’, minimum composition thresholds corresponding to amino acids in ‘-a’; ‘-w’, scanning window size; ‘-d’, linear dispersion threshold)

Domain type	Search parameters	# of Domains identified	Examples of identified domains	Protein source
Multifaceted prion-like domains	-a QN_Y -c 40_10 -w 60 -d 0.6	18	• QHRYMEGFSNNNNKQYRQNRNYNNNNNNSNNNHGSNYNNFNNGNSYIKGWNKNFNKYRRPSSSSY	• Ksp1
			• QQQQPQQQPAYYDIFGNPISQDEYLQYQYQQDQEQAMAQQRWLDQQQEQQQLAEQQYFQQQQQ	• Ent2
		
		
G/R/Y-rich domains associated with LLPS	-a G_RY -c 30_15 -w 60 -d 0.7	10	•GEYIDNRPVRLDFSSPRPNNDGGRGGSRGFGGRGGGRGGNRGFGGRGGARGGRGGFRPSGSGANTAPLGRSRNTASFAG	• Nsr1
			• GPPKPKNKKKRSGAPGGRGGASMGRGGSRGGFRGGRGGSSFRGGRGGSSFRGGSRGGSFRGGSRGGSRGGFRGGRR	• Gar1
		
		
Pab1-like P-rich LCDs	-a QN_P_G_ILMVF -c 15_15_10_10 -w 60 -d 0.5	52	• PRYYQPQQPQYPQYPQQQRYYPQQAPMPAAAPQQAYYGTAPSTSKGSGHGGAMMGGLLGVGAGLL	•Wwm1
			• QAQARQNQGTAPLNPYPGLTVTEPSFANPAGGYADGDLYPVGTSHPDWSGGLPNPLGNPSSQ	• Fub1
		
		
Highly charged domains (w/ high fraction of positively + negatively charged residues)	-a DE_KR -c 40_40 -w 30 -d 0.5	10	• EDEEKKKNEEEEKKKQEEKNKKNEDEEKKKQEEEEKKKNEEEEKKKQE	•Mnn4
			• EEEQKEEVKPEPKKSKKEKKRKHEEKEEEK	• Fpr3
			• KKRKREGDDSEDEDDDDKEDKDSDKKKHKKHKKHKKDKKKD	• Pxr1
		

### Exhaustive composition analyses illuminate a second layer of compositional and functional diversification among LCDs

**Figure 7.**
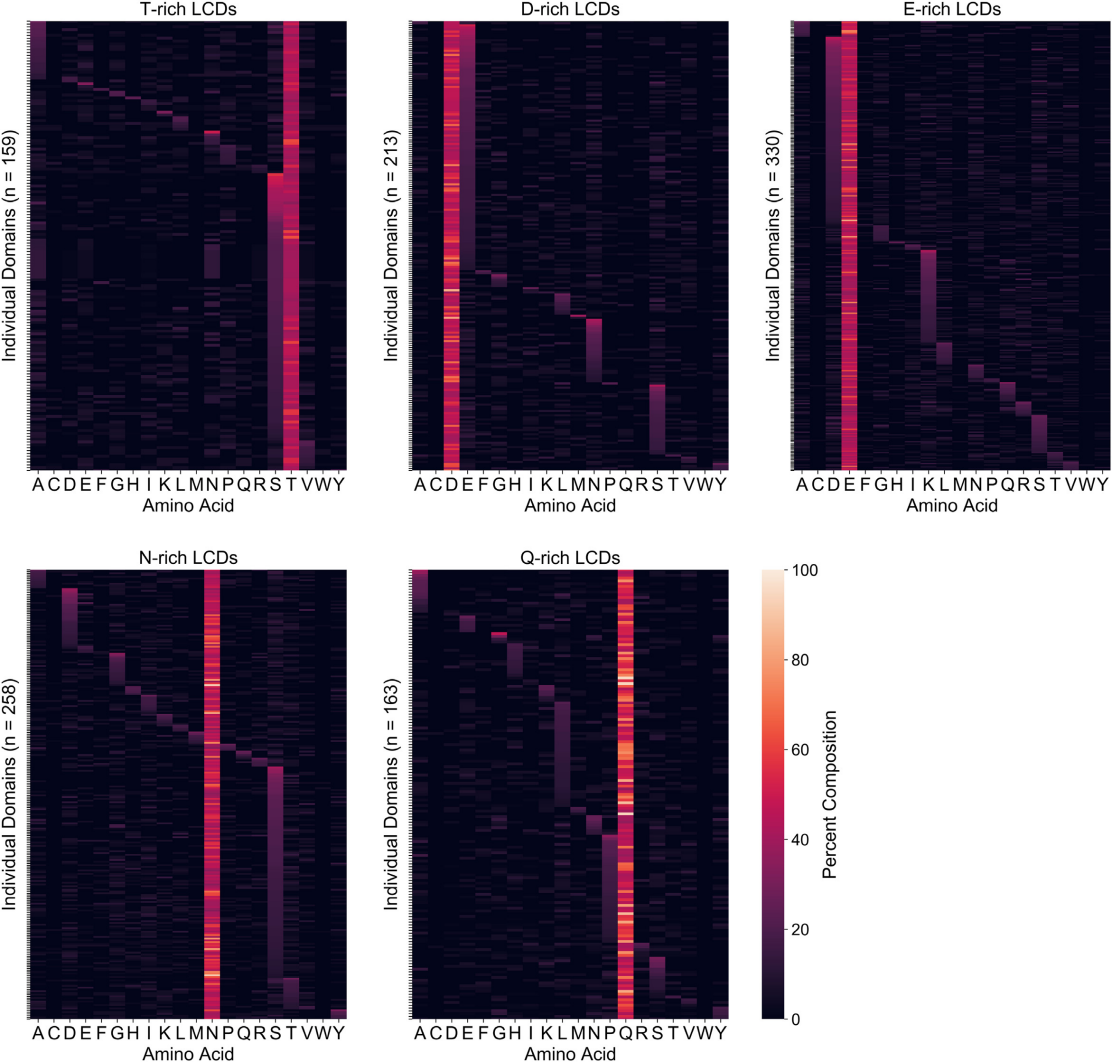
Yeast primary LCD classes exhibit unique preferences for secondary amino acids. Complete composition analyses were performed for all LCDs for which a secondary amino acid could be unambiguously assigned to a single residue type (i.e. a second amino acid with the next highest composition, excluding the primary amino acid). Heatmaps indicate percent composition of each amino acid (x-axis) for each LCD (y-axis), on a scale from 0–100%. Some classes of LCDs exhibit a strong preference for a single secondary amino acid (T-rich LCDs) or multiple secondary amino acid classes (D-rich, E-rich, N-rich and Q-rich LCDs), and secondary preferences observed for some primary LCD classes do not strongly overlap with those of related primary LCD classes (e.g. D-rich versus E-rich LCDs, and N-rich versus Q-rich LCDs). Complete composition analyses for the remaining LCD classes and model organisms are indicated in Supplementary Figures S13, S14 and Table S3).

**Figure 8.**
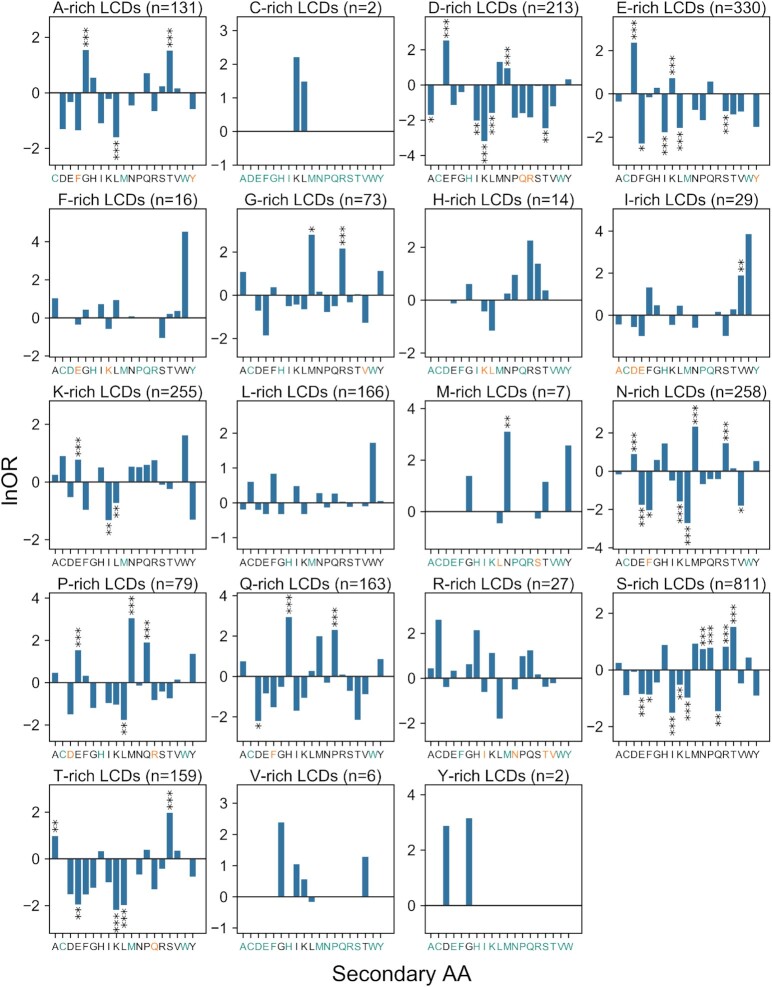
Quantitative analysis of secondary amino acid preferences among primary LCD classes. For each LCD class, the number of LCDs observed for each possible secondary amino acid was compared to corresponding window frequencies derived from a whole-proteome scan of the yeast proteome (see Materials and Methods). The natural log of the odds ratio (lnOR) indicates the degree of enrichment or depletion of LCDs with a secondary amino acid relative to whole-proteome frequencies (see Material and Methods section). Indications of statistical significance are from Bonferroni-corrected *P*-values (***P < 0.001, **P < 0.01, *P < 0.05, see Supplementary Table S8). Secondary amino acid categories with a scaled whole-proteome frequency <1 are colored teal to distinguish them from categories with a true lnOR = 0. For secondary amino acid categories with no observed LCDs (colored orange), an imputed observed value of 1 was used as a conservatively biased estimator.

Secondary compositional biases have been noted previously for specific classes of LCDs (8,9) but have not been thoroughly examined for all LCDs. Secondary biases among LCDs could, in principle, lead to subclasses of LCDs within each primary LCD class. To explore this possibility, the composition of all 20 canonical amino acids was calculated for each individual LCD identified by LCD-Composer within the yeast proteome. Indeed, many primary classes of LCDs exhibit strong preferences for a second amino acid resulting in clustered subclasses of LCDs (Figures 7 and 8, Supplementary Figures S13, S14 and Table S8). For some types of LCDs a single cluster is observed, indicating a strong secondary preference for only one type of amino acid (e.g. T-rich LCDs with a strong secondary preference for S; Figures 7, 8 and Supplementary Table S8). For other classes of LCDs multiple distinct clusters of varying sizes are observed (e.g. D-rich LCDs exhibiting secondary preferences for E, N or S, Figures 7, 8 and Supplementary Table S8), suggesting a partitioning of the primary LCDs into specialized subclasses. Strikingly, in many cases the secondary preferences are not strongly overlapping even for apparently similar classes of LCDs. For example, while both D-rich and E-rich LCDs exhibit secondary preferences for each other, E-rich LCDs contain a cluster of LCDs secondarily enriched in K, whereas D-rich LCDs are almost completely devoid of secondary enrichment for K (Figures 7, 8 and Supplementary Table S8). Similarly, N-rich LCDs exhibit secondary preferences for D or S, while Q-rich LCDs exhibit secondary preferences for H, L or P (Figures 7, 8 and Supplementary Table S8).

**Figure 9.**
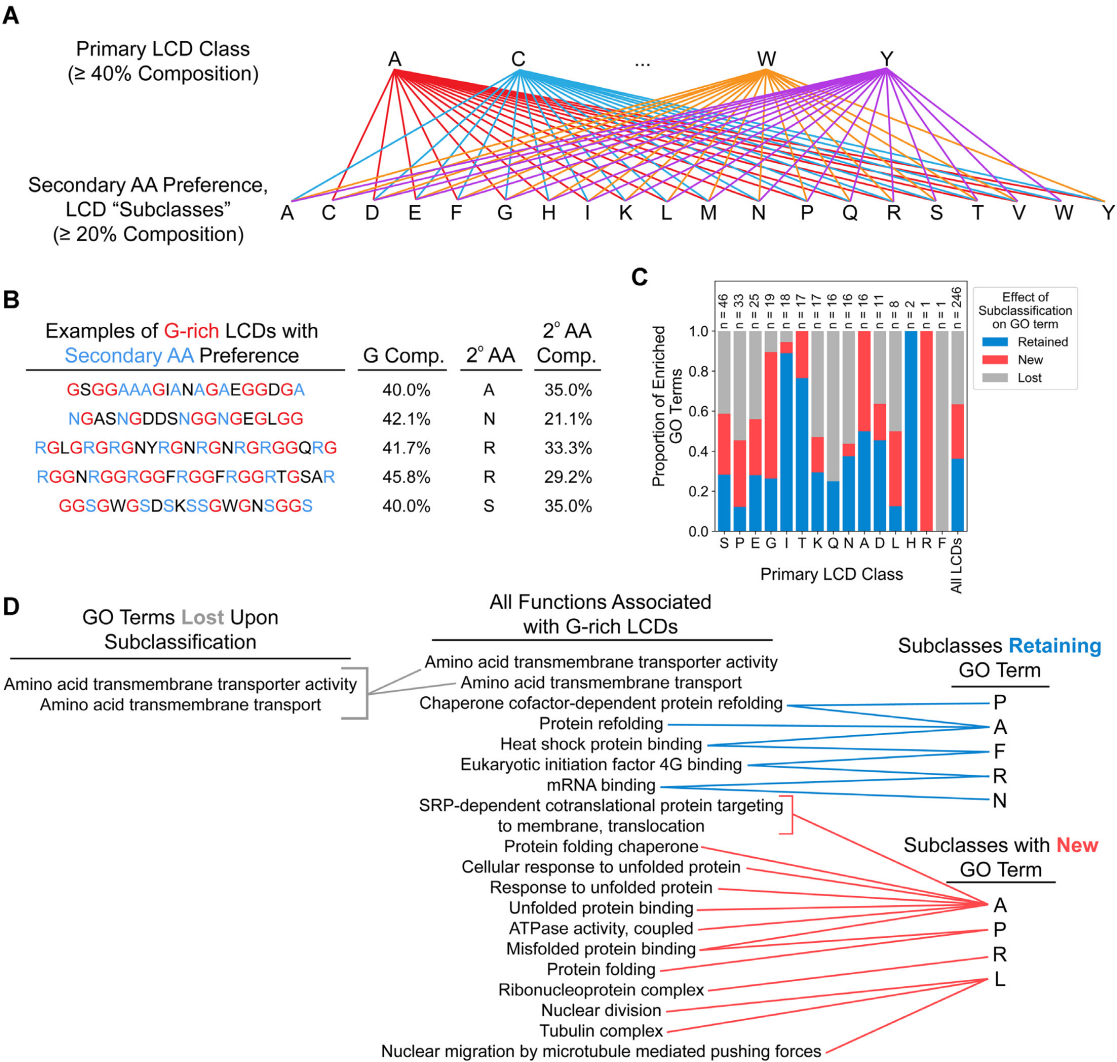
The effects of subclassification on GO term retention, loss, or gain reveal a second layer of functional diversification among yeast LCDs. (**A**) Multifaceted LCD-Composer search criteria were used to identify LCDs for each possible LCD subclass (≥ 40% composition for a primary amino acid and ≥ 20% composition for a secondary amino acid). (**B**) Example of diverse G-rich domains with differing secondary amino acids and secondary amino acid compositions. (**C**) For each primary LCD class, the proportions of GO terms retained, lost, and new upon subclassification are indicated as stacked bars. (**D**) Complete GO term retention, loss, and *de novo* appearance (‘new’) network for yeast G-rich LCDs. Full results for all LCD subclasses across all model organisms are available in Tables S9 and S10.

These observations suggested that particular subclasses of LCDs emerge due to functional specialization within each primary LCD class. Therefore, we re-analyzed the yeast proteome with LCD-Composer using the built-in capacity for specifying multifaceted composition criteria. Specifically, for each of the 20 canonical amino acids, the yeast proteome was searched for all LCDs with at least 40% composition of the primary amino acid and at least 20% of a secondary amino acid (Figure 9A,B), resulting in 380 possible pairwise search combinations (each of the 20 primary amino acids by each of the 19 possible secondary amino acids). GO term analyses were performed for each set of identified LCDs, which we refer to as LCD ‘subclasses’. *A priori*, we expected three possible outcomes. First, a GO term may co-segregate with specific subclasses of LCDs (i.e. the GO term is ‘retained’ by at least one subclass), suggesting that the original enrichment observed may actually be attributable to a specialized subset among the larger LCD class. Second, a functional annotation might be enriched for the primary LCD class as a whole but ‘lost’ among the LCD subclasses, likely due to a reduction in sample size or to the contribution of multiple LCD subclasses to the original enrichment. Finally, ‘new’ GO term annotations may appear for specific subclasses of LCDs if those LCDs (and not other subclasses of LCDs) fulfill a specialized functional role in the cell (effectively modulating the ‘signal-to-noise’ ratio via retention of relevant LCD subclasses and exclusion of irrelevant subclasses).

GO term retention, loss, and *de novo* appearance was determined for all primary LCD classes and secondary LCD subclasses across all seven eukaryotic organisms. Functional annotations for nearly all primary classes of LCDs exhibit each of the three possible effects resulting from subclassification (retention, loss, and *de novo* appearance), though to varying degrees across LCD class and organism (Supplementary Table S9). For example, S-rich LCDs in yeast are associated with roughly equal proportions of retained, new, and lost GO terms, while A-rich LCDs are associated with equal proportions of new and retained GO terms (Figure 9C and Supplementary Table S9). Proteins with G-rich LCDs exhibit the highest proportion of new GO terms (excepting R, which only had one associated GO term), though all three possible subclassification effects are observed (Figure 9C). For example, amino acid transport functions associated with the primary class of G-rich domains in the yeast proteome do not appear in any of the subclasses, so these annotations were lost upon subclassification (Figure 9D). However, multiple annotations related to protein folding and protein chaperone activity are maintained or new across certain subclasses (namely, G-rich LCDs with a secondary preference for A, F or P). Similarly, functions related to mRNA-binding, ribonucleoprotein complexes, and translation initiation factor binding are specifically maintained by G-rich LCDs with a secondary preference for N, R or F residues. Finally, multiple functional annotations related to tubulin, microtubules, and microtubule-mediated nuclear migration are specifically associated with G-rich LCDs with a secondary preference for L residues, even though these functions were not detected as enriched among G-rich domains generally (i.e. *de novo* appearance only upon subclassification). Notably, the majority of GO terms associated with most LCD classes are still detected when highly homologous proteins within each LCD class are excluded (Supplementary Figure S15A, B). Additionally, the log-odds ratios indicating the degree of GO term enrichment for subclassified LCD protein sets is nearly always greater than that of primary LCD protein sets and, in many cases, with non-overlapping confidence intervals (Supplementary Table S10), indicating that LCD subclassification specifically and broadly enhances enrichment of functional annotations.

In summary, the composition-centric approach employed by LCD-Composer illustrates the diversity of LCDs within and across eukaryotic organisms, and enables finer, multi-layered classification of LCDs.

### Tracking co-occurrence of distinct classes of LCDs within individual proteins reveals functional associations for multi-LCD proteins

**Figure 10.**
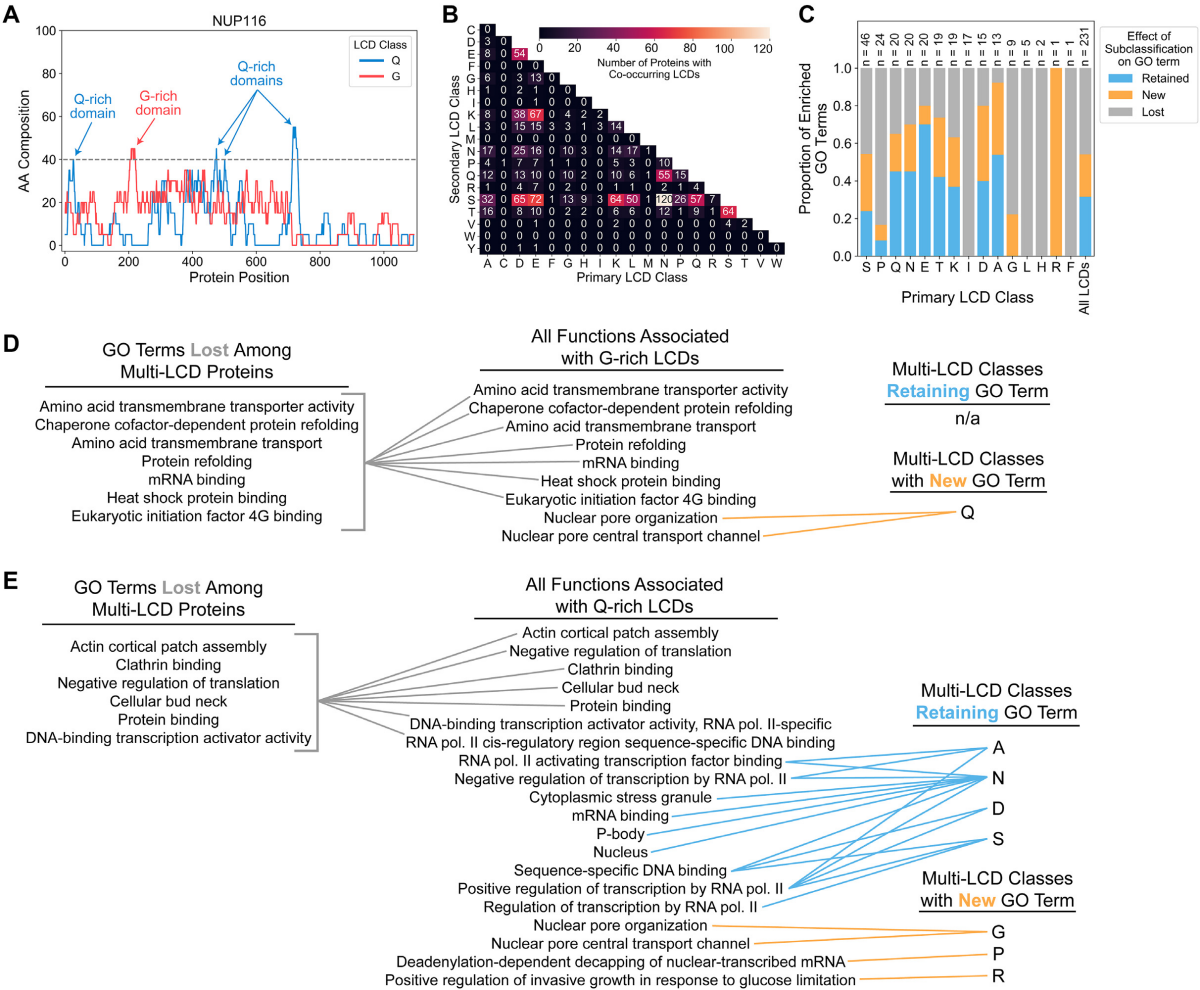
GO term retention, loss, or gain as a result of LCD co-occurrence indicates shared and unique functions of multi-LCD proteins. (**A**) The Nup116 protein contains non-overlapping G-rich and Q-rich LCDs. (**B**) Heatmap depicting the number of instances of co-occurring LCDs for each LCD class. The upper-right half is numerically equivalent and therefore omitted for simplicity. (**C**) For each primary LCD class, proteins with at least one additional non-overlapping LCD were sorted into each co-occurring LCD secondary class. Each secondary class was then evaluated for significantly enriched functional associations. The resulting proportions of GO terms retained, lost, and new for multi-LCD proteins are indicated as stacked bars. (**D**) Complete GO term retention, loss, and new network for yeast G-rich multi-LCD proteins. (**E**) The complete GO network for yeast Q-rich multi-LCD proteins demonstrates reciprocal *de novo* appearance of the new GO terms associated with G-rich multi-LCD proteins. Q-rich multi-LCD proteins also exhibit additional class-specific retention, loss, and gain of GO terms. Full results for all multi-LCD proteins across all model organisms are available in Tables S11 and S12.

As depicted in Figure 3B, some proteins contain multiple non-overlapping LCDs from distinct LCD classes. This raises the intriguing possibility that proteins with multiple concurrent LCDs (e.g. proteins with both a G-rich domain and a Q-rich domain; Figure 10A) could specifically participate in particular functions that are not associated with the LCD classes individually. A number of yeast proteins contain non-overlapping LCDs of distinct LCD classes (Figure 10B). For each LCD class, multi-LCD proteins were further parsed into separate classes based on the predominant residue of each additional non-overlapping LCD. GO term analyses were then performed separately for each set of parsed multi-LCD proteins. Most primary LCD classes exhibit a mixture of GO term loss, retention, and *de novo* appearance upon multi-LCD sorting (Figure 10C and Supplementary Table S11). For G-rich LCDs, the majority of GO terms are lost when LCDs are divided among co-occurring LCD categories, likely due to smaller sample sizes associated with dual enrichment. However, proteins with non-overlapping G-rich and Q-rich LCDs are associated with nuclear pore organization and transport functions (Figure 10D). Importantly, these functional associations are also new GO terms when Q-rich LCDs are considered as the primary class (Figure 10E), indicating that these functions are specifically associated with the subset of LCDs containing both G-rich and Q-rich LCD classes (and not the individual LCD classes). Enriched GO term associations were not due to highly homologous proteins within each LCD class (Supplementary Figure S15C). Again, the degree of GO term enrichment for multi-LCD protein sets was nearly always greater than the degree of GO term enrichment for the original primary LCD protein sets (Supplementary Table S12). Together, this suggests that proteins containing specific combinations of non-overlapping LCDs may also fulfill specialized molecular roles.

## DISCUSSION

Recent studies have suggested that the amino acid composition and linear dispersion of amino acids within LCDs are important—if not predominant—features governing their biophysical behavior (15,41,64–68). LCD-Composer was developed with this emerging view in mind. Although a variety of methods exist for identifying LCDs in proteins, the central focus of LCD-Composer is the amino acid composition of LCDs, making it intuitive to biologists and relevant to the actual physicochemical properties of the identified LCDs. While primary sequence undoubtedly plays a role in the functional properties of some LCDs, methods designed for initial classification of LCDs are an important step before more nuanced classification on the basis of specific features. In the future, integration of additional information including post-translational modifications, short linear motifs, intrinsic disorder, repetitiveness, and related features may lead to a richer LCD classification system.

LCD-Composer was designed specifically for the identification of LCDs on the basis of customizable composition profiles, irrespective of whole-proteome amino acid frequencies. In contrast to existing methods relying on mathematical sequence complexity or statistical enrichment of amino acids, LCD-Composer's composition-based approach is extremely flexible, intuitive to use, and generates results that are easy for the average user to interpret. The simplicity of the LCD-Composer method and search parameters enables multifaceted LCD search criteria, including user-defined groupings of amino acids and distinct composition thresholds for each amino acid or group of amino acids, which cannot be easily implemented with existing methods.

The speed and specificity of LCD-Composer make it a powerful yet intuitive LCD-identification method. Our database of simple LCDs identified for each LCD class across all organisms available from UniProt should serve as a valuable resource for researchers interested in specific types of LCDs. However, we would like to emphasize that these LCDs are derived from only one set of search criteria, even though LCD-Composer allows for an infinite number of combinations of amino acid(s) of interest, window size, composition threshold(s), and dispersion threshold. Therefore, LCD-Composer may still be of great utility to users wishing to specify non-default or composite search parameters.

Our survey of LCDs in all reference proteomes raises a number of interesting and currently unanswered questions. The proteomes of *Dictyostelium discoideum* and *Plasmodium falciparum* were already known to have extremely high Q/N-rich and N-rich LCD content, respectively, and exhibit corresponding adaptations in proteostasis machinery that specifically accommodate such a high prevalence of aggregation-prone domains (69,70). However, our database of LCDs unveils a multitude of additional organisms with unusually high LCD content for specific classes of LCDs (even typically rare types of LCDs). For example, M-rich domains constitute ∼0.75% of the proteome of the intestinal parasite, *Echinostoma caproni* (compared to ∼0.006% average M-rich content among eukaryotes), while H-rich domains constitute nearly 2% of the *Spodoptera litura* (Asian cotton leafworm) proteome. How might these organisms have adapted to such an unusually high prevalence of particular LCDs or, conversely, how might prior adaptations have facilitated the development and utilization of these LCDs? What are the implications for protein synthesis, folding, and degradation systems in these organisms, and how do these systems differ across organisms with extremely high LCD content for different LCD classes? Are these adaptations specific to certain ecological niches? How might the discovery of new proteostasis machinery or mechanisms aid in the development of new biotechnology or human disease therapeutics? LCD-Composer and our database of LCDs provide a valuable launchpad for exploring these questions in both model and non-model organisms.

LCD-Composer's customizable search parameters enable specific and selective LCD searches. We demonstrate that these features can be used to resolve LCDs into richer hierarchies on the basis of multiple compositional features, including LCD subclasses (enriched in more than one amino acid) and co-occurring LCDs (non-overlapping LCDs in the same protein). Each level of the hierarchy appears to be of functional importance: in many cases, primary LCD classes were associated with particular functions that were lost upon subclassification, while other functional associations were only detected after subclassification. Therefore, integrating both fine and coarse resolution of LCDs yields a more complete picture of LCD functional specificity and diversity. However, it is also worth noting that some LCDs may exist for reasons unrelated to protein function, such as genomic nucleotide composition or non-functional repeat expansion. While GO term analyses can unveil statistical relationships between LCD classes and associated LCD functions, definitive assignment of functions (or lack thereof) to specific LCDs identified by LCD-Composer should be determined experimentally. Finally, particular classes of LCDs fulfill similar or identical molecular roles across a broad range of model eukaryotes, suggesting that the unusual sequence features of LCDs may occupy molecular niches and are indispensable for certain molecular processes. In our view, the combination of simplicity, flexibility, and direct quantification of biochemically relevant LCD features make LCD-Composer a powerful, intuitive, and adaptable tool for protein research.

## DATA AVAILABILITY

The LCD-Composer script and detailed usage information are available at https://github.com/RossLabCSU/LCD-Composer. All code required to fully reproduce the data presented in this paper are available at https://github.com/RossLabCSU/LCD-Composer/tree/master/Reproducibility. Databases of all simple LCDs identified using LCD-Composer's default parameters for all available reference proteomes from UniProt are available at (46).

## Supplementary Material

lqab048_Supplemental_Files

## Notes

Present address: Department of Biochemistry and Molecular Biology, 1870 Campus Delivery, Colorado State University, Fort Collins, CO 80523, USA.
