# Supplementary material for: LCD-Composer: an intuitive, composition-centric method enabling the identification and detailed functional mapping of low-complexity domains
Source: NAR Genom Bioinform. 2021 May 26;3(2):lqab048. doi: 10.1093/nargab/lqab048 (PMC8153834; doi:10.1093/nargab/lqab048)
Supplement: lqab048_Supplemental_Files [file lqab048_supplemental_files.zip › Supplemental_Material_File_NARGAB_3rd_Resubmission.zip!/Supplemental Material File_NARGAB_3rd Resubmission.docx]

**Supplementary Material**

**Table of Contents**

[Supplementary Discussion, Figs S1-S6	2](#_Toc66192866)

[Development and Testing of the Linear Dispersion Parameter for Defining LCD Boundaries	2](#_Toc66192867)

[Systematic Evaluation of the Effects of Varying LCD-Composer Parameters on LCD Identification and Definition	4](#_Toc66192868)

[Window Size	4](#_Toc66192869)

[Linear Dispersion	7](#_Toc66192870)

[Composition Threshold	7](#_Toc66192871)

[LCD-Composer Performance is Fast, Scalable, and Relatively Insensitive to User-Defined Parameters	7](#_Toc66192872)

[Fig S7	10](#_Toc66192873)

[Fig S8	11](#_Toc66192874)

[Fig S9	12](#_Toc66192875)

[Fig S10	13](#_Toc66192876)

[Fig S11	14](#_Toc66192877)

[Fig S12	16](#_Toc66192878)

[Fig S13	17](#_Toc66192879)

[Fig S14	18](#_Toc66192880)

[Fig S15	19](#_Toc66192881)

[Supplementary Table Legends	20](#_Toc66192882)

[References	22](#_Toc66192883)

Supplementary Discussion, Figs S1-S6

Development and Testing of the Linear Dispersion Parameter for Defining LCD Boundaries

The following is an extensive discussion and analysis of the linear dispersion parameter developed for LCD-Composer definition of LCD boundaries, which is intended to serve as a guide for users wishing to specify a custom linear dispersion threshold when running LCD-Composer.

In order to develop a linear dispersion parameter suitable for evaluating amino acid spacing within LCD sequences, we generated an exhaustive set of benchmark sequences 20 residues in length and consisting of only two characters (G and X, simulating the evaluation of G spacing vis-à-vis all other residues, represented simply as X). Specifically, all possible sequence permutations were generated at every possible G composition level resulting in 1048574 unique sequences. For a given protein sequence window, the spacings of all residues of interest (in this case, G) from each other as well as the window termini are calculated. This procedure is repeated to calculate the spacing of all other residues from each other and from the window termini. All spacing values are then combined into a single array, and the standard deviation is calculated, normalized based on the minimum and maximum possible standard deviation (see Fig 1 and Material and Methods), then subtracted from 1, resulting in the final linear dispersion value. The linear dispersion parameter was also validated on an exhaustive set of benchmark sequences for all sequence lengths between 5 and 30 residues (inclusive), and should be effective for all other sequence lengths as well. Users interested in further confirming determination of the linear dispersion parameter are referred to https://github.com/RossLabCSU/LCD-Composer/tree/master/Reproducibility/LinearDispersionDevelopment.

Linear dispersion values for all benchmark sequences can be generated by following the instructions at the Github link above. An effective linear dispersion parameter should be stable across a range of compositions and window sizes. We first evaluated the distributions of linear dispersion values when parsed by the number of mutations (Fig S1). Linear dispersion distributions tend to be centered ~0.7 (median) with skew toward 0 for all composition classes, suggesting that linear dispersion is relatively stable regardless of window sequence composition (Fig S1; “Number of Mutations”, indicating the number of X’s among each sequence). To evaluate a possible dependence of linear dispersion discrimination across composition classes, LCD-Composer was run on the set of benchmark sequences while varying the linear dispersion threshold. The percentage of sequences that would be excluded at each linear dispersion threshold for each composition class was then calculated and plotted. Curves for each composition class are generally sigmoidal with similar shapes and midpoint values (Fig S2). All curves have a minimum of 0% of sequences excluded at a linear dispersion threshold=0.0, and approach (but do not reach) 100% of sequences excluded at a linear dispersion threshold=1.0 since at least one perfectly dispersed sequence exists in every composition class. The smallest two classes exhibit more discrete behavior due to the low number of possible sequence permutations and fewer spacing values that contribute to the standard deviation. However, it is important to note that the effect of slight differences between curves is mitigated in the context within which the linear dispersion parameter is used. Specifically, LCD-Composer scans protein sequences linearly and merges overlapping windows that pass the composition and dispersion criteria. As LCD-Composer reaches an LCD region, the distribution of the amino acid(s) of interest are often asymmetrically distributed, resulting in low linear dispersion values. However, as the sliding window progresses, more of the amino acids of interest are incorporated into the frame and become more centralized, resulting in higher linear dispersion values. LCD-Composer then merges overlapping windows that pass the composition and linear dispersion criteria, effectively bridging across short sequence stretches with an asymmetric distribution of the amino acid of interest, provided the sequence is sufficiently dispersed to pass the linear dispersion threshold when the sliding window places the intervening residues are in the most dispersed and centralized positions possible.

**Fig S1. Linear dispersion distributions are similar across the full range of composition classes.** For each composition class (“Number of Mutations”) the distribution of linear dispersion values for all benchmark sequences is indicated as a violin plot.

**Fig S2. Discriminatory effect of the linear dispersion threshold on benchmark sequences across composition classes.** For each composition class (“Number of Mutations”) the percentage of sequences with linear dispersion values below the threshold was calculated for a defined series of linear dispersion thresholds (0.0-1.0, in increments of 0.1) and plotted. Sequences with linear dispersion values below the threshold would be “excluded” by LCD-Composer.

Fig S3 demonstrates the effect of intervening gaps as a function of linear dispersion threshold. In the absence of a linear dispersion value (i.e., linear dispersion threshold = 0.0), all overlapping windows with compositions exceeding the specified composition threshold are merged regardless of the distribution of the amino acid of interest. As the linear dispersion threshold is increased, the number of adjacent intervening residues required to split nearby LCDs into two separate domains gradually decreases until a threshold of 0.7. At high linear dispersion thresholds (>0.7), two adjacent intervening residues are sufficient to split nearby LCDs into two distinct LCDs. A single intervening residue is insufficient for splitting LCDs, since at least one overlapping window contains the intervening residue at the central (perfectly dispersed) position. Therefore, although the curves for percentage of benchmark sequences excluded as a function of linear dispersion for the single or double mutation sets are most dissimilar to the curves for other composition classes, a single intervening residue will not ultimately impact LCD bridging at any linear dispersion threshold, and two intervening residues will only affect LCD bridging in the rare cases in which a very high linear dispersion threshold is specified and the two residues are nearly (or truly) adjacent. However, even in such cases, nearby LCDs will simply be identified as two distinct domains.

**Fig S3. Effects of the linear dispersion threshold on defining nearby LCDs.** A set of benchmark sequences consisting of long G-rich regions disrupted by an intervening cluster of between 1-20 unrelated residues was evaluated by LCD-Composer at various linear dispersion thresholds. For each linear dispersion threshold, the minimum number of mutations sufficient to split the identified G-rich LCD into two distinct domains was calculated and plotted.

In summary, the linear dispersion parameter is robust across a range of compositions and constitutes a customizable feature that discriminates well-mixed/dispersed sequences from asymmetrically-distributed/clustered sequences.

Systematic Evaluation of the Effects of Varying LCD-Composer Parameters on LCD Identification and Definition

To provide extensive user guidance, we analyzed the yeast proteome with each of the parameters varied independently, with all other parameters fixed as the default values (chosen based on initial exploratory analyses, and discussed below as well). Fig S4A-C shows the number of domains identified for each amino acid as the window size, minimum composition threshold, or linear dispersion threshold, respectively, are varied. Fig S5A-C show the effect of varying the window size, minimum composition threshold, or linear dispersion threshold on the average length of the domains identified. Each parameter is discussed in greater detail below.

Window Size

The choice of scanning window size will affect both the minimum domain length required for identification and the merging of neighboring windows. Short windows will generally lead to the identification of more regions passing the composition criteria for two main reasons. The first is that it is

statistically more likely that short regions (e.g. 10aa segments) achieve a relatively high composition of a particular amino acid when compared to long regions [e.g. 100aa segments; (1)]. Second, short windows will result in fewer identified regions being merged into a single domain, since the probability of overlapping regions passing the user-specified criteria will decrease as window size decreases. Although LCD-Composer is capable of extending overlapping domains indefinitely, running LCD-Composer using a short window size tends to result in a greater number of identified regions (Fig S4A) that have smaller average lengths (Fig S5A). Therefore, small window sizes will favor analyses conceptually-dependent on local compositional context [e.g. the local structural tendencies of LCDs (2), or identification of compositionally-skewed short linear motifs (3)], while longer windows naturally favor identification of large LCDs or mild compositional biases over large protein regions.

**Fig S4. Relationships between optional parameters and the number of LCDs identified.** The yeast proteome was evaluated using each of the 20 canonical amino acids while varying the scanning window size (A), linear dispersion threshold (B), and minimum composition threshold (C). For panels A and B, values in each cell (along with corresponding heatmap colors) indicate the number of LCDs identified for each indicated parameter constraint. For panel C, values indicate the fraction of the proteome classified as LCD. Heatmap colors are on a log scale normalized within each heatmap to visually emphasize differences at more stringent thresholds (higher values for all parameters varied). For each varied parameter, all other parameters are set at their default values.

**Fig S5. Relationships between optional parameters and the average lengths of LCDs identified.** For each of the analyses described for Fig S4, the average length of domains identified by LCD-Composer while varying search parameters was calculated and plotted in grouped bar charts. Increasing window size typically increases average domain length (A), while increasing the linear dispersion threshold tends to decrease average domain length (B). Varying the composition has mixed effects on average domain length (C).

Linear Dispersion

The linear dispersion threshold defines how well-mixed sequences must be before identification by LCD-Composer. At the high extreme, setting the linear dispersion threshold to 1.0 requires that sequences be perfectly well-mixed to be identified. At the low extreme, with a linear threshold of 0.0, sequences can be perfectly segregated and still be identified by LCD-Composer. To illustrate the relationship between the linear dispersion threshold and the stringency of LCD identification, LCD-Composer was run on the yeast proteome for each amino acid using a range of linear dispersion thresholds, with the minimum percent composition threshold fixed at 40% (the default composition threshold in LCD-Composer). For all amino acids, the number of LCDs identified is highest at or near a linear dispersion threshold of 0.0 (most permissive threshold) due to the inclusion of LCDs that pass the composition criteria even though the amino acid of interest may be clustered near the sequence terminus (Fig S4B). The number of LCDs typically decreases as the maximum linear dispersion threshold decreases, reflecting the fact that fewer segments contain sufficiently uniform amino acid spacing. Domains also tend to be shorter in length, since fewer neighboring regions are sufficiently spaced to pass the linear dispersion criteria and be merged into the final domain (Fig S5B), although the average length of domains can be spuriously large at extremely high linear dispersion thresholds (where sample size is lowest) or for amino acids with a small number of LCDs.

Composition Threshold

Amino acid composition is the predominant parameter affecting the number and characteristics of identified domains. The simplest LCD-Composer searches identify domains on the basis of a single type of amino acid. As expected, low minimum composition thresholds result in the classification of a large fraction of the proteome as regions of interest (Fig S4C). When the results are sorted based on the number of domains identified at the lowest composition threshold (10%), the rank order of the amino acids almost perfectly recapitulates the rank order of amino acids based on their simple whole-proteome frequencies, suggesting that these results largely reflect whole-proteome composition characteristics rather than preferentially identifying individual domains with unusual compositions. However, as the minimum composition threshold is increased, the fraction of the proteome classified as regions of interest decline substantially, and the total number of domains identified for the amino acids differ notably from whole-proteome amino acid frequencies. These changes become most apparent near the 40% minimum composition threshold. For example, L, S, and K are the three most abundant amino acids in the yeast proteome and have the highest fraction of the proteome identified with the 10-30% composition thresholds (Fig S4C is sorted based on results for the 10% composition threshold). However, the fraction of the proteome classified as S-rich domains at a 30% composition threshold is much larger than that of L-rich or K-rich domains, and this disparity increases as the composition threshold is increased, which is consistent with previous analyses (1). Conversely, Q is the 6^th^ rarest amino acid in the yeast proteome, but the number of Q-rich domains identified at the 40% composition threshold is the 6^th^ highest among the amino acids and 2^nd^ highest at the 50% composition threshold. Based on these analyses, the 40% composition threshold was chosen as the default for identifying LCDs, although we recommend that users consider specifying alternative composition thresholds depending on the type of LCD of interest and the compositional characteristics of the proteome to be analyzed. For certain amino acids (N, Q, and S), moderate composition thresholds led to the largest final domain lengths, with lower domain lengths occurring at both extremes of the composition threshold spectrum (Fig S5C). For others (A, D, G, H, K, T, W, and Y) there is a slight upward trend toward longer LCDs at higher composition thresholds. For the remaining amino acids, no discernible domain length pattern emerges as the composition threshold is varied.

LCD-Composer Performance is Fast, Scalable, and Relatively Insensitive to User-Defined Parameters

To further evaluate the performance of LCD-Composer, we measured the computation speed of LCD-Composer on a simple desktop computer for all benchmark tests on the yeast proteome described above (see Material and Methods). First, we explored the effect of varying the three main search parameters (composition threshold, linear dispersion threshold, and window size) on LCD-Composer speed. Extremely low composition thresholds (10% and 20%) largely reflect whole-proteome amino acid frequencies and are unlikely to be useful when running LCD-Composer (Fig S4C and associated discussion above), so tests employing these thresholds were excluded from analyses. The mean computation time across all parameters for the yeast proteome is ~4.0sec. Computation time is only weakly influenced by amino acid of interest, composition threshold, window size, and linear dispersion threshold (Fig S6A), indicating that LCD-Composer speed is not substantially impacted by variations in user-defined parameters (although we expect computation time to increase linearly with window size, so times may increase noticeably for very large window sizes).

Next, we explored the scalability of LCD-Composer by measuring the time required to evaluate proteomes of diverse sizes corresponding to common model organisms. As expected, computation time increases for larger proteomes (Fig S6B), though the maximum computation times for the largest proteomes evaluated are ~32.4sec (*D. rerio*) and ~33.1sec (*H. sapiens*), and LCD-Composer computation time scales linearly with proteome size (Fig S6C).

Collectively, these results show that LCD-Composer is amenable to high-throughput analyses involving single or multiple proteomes across a wide variety of possible search parameters.

**Fig S6. LCD-Composer is fast, relatively insensitive to specified parameters, and scales linearly with proteome size.** (A) The yeast proteome was evaluated with LCD-Composer using all possible combinations of amino acid of interest and: 1) composition thresholds in the range 20-100% in increments of 10, 2) linear dispersion thresholds in the range 0.1-1.0 in increments of 0.1, and 3) window sizes in the range 10-100 in increments of 10. Each bar indicates the mean computation time (in seconds) and standard deviation across the linear dispersion threshold range for each amino acid. (B) LCD-Composer average computation time (in seconds) and standard deviation for each of the model organism proteomes, across all LCD classes searched for. (C) The mean computation time across all LCD searches for each organism increases linearly with total proteome size (total number of amino acids).

Fig S7

**Fig S7. Cross-organism GO term overlap is specific for homotypic LCD category pairing across all organism comparisons.** (A) Cartoon depiction of the method used to estimate the specificity of GO term overlap for homotypic (e.g. *S. cerevisiae* A-rich LCDs vs. *C. elegans* A-rich LCDs) as opposed to heterotypic (e.g. *S. cerevisiae* A-rich LCDs vs. *C. elegans* D-rich LCDs) LCD categories across organisms. For all possible organism pairs the proportion of GO terms for each LCD category for the primary organism found in each of the LCD categories of the secondary organism was calculated and plotted as a heatmap. (B) Observed mean proportion of overlap in GO terms for on-diagonal and off-diagonal squares for each organism pair. In all cases the difference between on-diagonal and off-diagonal values is highly significant (*P* < 0.001, two-sided Mann-Whitney *U* test), with one exception (*X.* *laevis* + *S. cerevisiae*, where *P*=0.0012).

Fig S8

**Fig S8. Observed cross-organism GO term counts compared with iterative, length-weighted protein sampling.** In principle, longer proteins could be disproportionately associated with particular GO annotations and also have a higher probability of containing one or more LCDs. To account for possible confounding effects due to protein length, proteins were randomly sampled without replacement for each organism and LCD class until the sample size matched that of the observed number of proteins in the LCD class for that organism. Protein sampling was weighted by protein length, such that longer proteins were proportionally more likely to be sampled. GO term analyses were separately performed for each set of proteins. For all significantly enriched GO terms (Šidák-corrected *P* < 0.05) across the 7 organisms, the number of organisms sharing an identical GO term was calculated. This process was repeated for 1000 iterations for each LCD class. Red bars indicate the mean count (± s.d.). Blue bars indicate observed cross-organism GO term counts and are identical to those in Fig 6.

Fig S9

**Fig S9. Observed cross-organism GO term counts when all protein isoforms are included in analyses.** GO term annotations are assigned to gene products (not genes), raising the possibility that isoforms with LCDs are linked to specific GO annotations compared with those lacking LCDs, or vice versa. Therefore, we repeated the GO term analyses as indicated for Fig 6 using proteomes containing all known isoforms for each organism (also downloaded from Uniprot). Blue bars indicate observed cross-organism GO term counts.

Fig S10

**Fig S10. Observed cross-organism GO term counts when sequence homology-based, automatically assigned GO annotations are excluded.** All GO annotations assigned to gene products are associated with an evidence code indicating the source or method of assigning the GO annotation. In principle, GO annotations assigned on the basis of sequence homology could bias cross-organism analyses of GO term enrichment and spuriously increase the number of significantly enriched GO terms detected. To account for this effect, all GO annotations with evidence codes “Inferred from Sequence or Structural Similarity” (ISS), “Inferred from Sequence Orthology” (ISO), “Inferred from Sequence Alignment” (ISA), “Inferred from Sequence Model” (ISM), and “Inferred from Electronic Annotation” (IEA) were removed from the gene annotation files. GO analyses for LCD-containing proteins were repeated as indicated for Fig 6 using the filtered gene annotation files. *X. laevis* was excluded from this analysis due to an extremely high proportion of total GO annotations assigned on the basis of the excluded evidence codes. Blue bars indicate observed cross-organism GO term counts.

Fig S11

**Fig S11. Statistical comparison of observed cross-organism GO term counts compared with iterative GO term sampling.** To estimate the frequency with which identical GO terms would occur in multiple organisms by chance (assuming a GO term sample size equal to the number of enriched GO terms for an LCD class), GO terms were iteratively and randomly selected without replacement for each organism and LCD class until the GO term sample size matched the sample size of the enriched GO terms for the given LCD class and organism. Importantly, since GO terms with few associated proteins may not be detected as statistically significant even if all associated proteins are present in an LCD class, we determined the minimum number of proteins associated with a GO term to be detected as statistically significant for each combination of LCD class and organism, and excluded GO terms with fewer associated proteins prior to GO term sampling. This process was repeated for a total of 100k iterations. For each iteration, the cross-organism counts of all GO terms were calculated. All bars indicate the observed cross-organism GO term counts as shown in Fig 6. Observed cross-organism GO term counts were then statistically compared to the cross-organism GO term counts derived from random sampling using a two-sided Fisher’s exact test, with Bonferroni correction for multiple hypothesis testing applied within each LCD class (7 possible cross-organism count categories for each LCD class). Specifically, the odds ratio of observed to sampled cross-organism GO term counts was calculated for each cross-organism count category within each LCD class as:

$${OR}_{c}= \left( \frac{n_{c_{obs}}}{n_{{tot}_{obs}}-n_{c_{obs}}} \right)/\left( \frac{n_{c_{samp}}}{n_{{tot}_{samp}}-n_{c_{samp}}} \right)$$

where *n_totobs_* represents the total number of GO terms enriched for a specific LCD class, *n_cobs_* represents the number of observed GO terms enriched for the same LCD class for a specific cross-organism count category (*c*), *n_totsamp_* represents the total number of GO terms randomly sampled for a specific LCD class, *n_csamp_* represents the number of randomly sampled GO terms in *c*. All indications of statistical significance represent Bonferroni-corrected *P*-values (*P* < 0.001, “***”; *P* < 0.01, “**”; *P* < 0.05, “*”; see Tables S4, S5, and S7 for all underlying data).

Fig S12

**Fig S12. Counts of GO terms associated with each LCD class across 7 model eukaryotic organisms.** For each LCD class, all significantly enriched GO terms (Šidák-corrected *P* < 0.05) identified across the 7 model eukaryotic organisms were combined into a single pool. Identified GO terms were then parsed into separate categories based on the number of organisms with significant enrichment for each GO term (Fig 6 and Tables S4 and S5). The proportion of combined GO terms for each LCD class associated with each cross-organism count category is indicated as a stacked barchart.

Fig S13

**Fig S13. Comprehensive composition analyses for A-, C-, F-, G-, H-, I-, K-, M-, P-, R-, V-, and Y-rich LCDs in yeast.** Complete composition analyses were performed as described for Fig 7.

Fig S14

**Fig S14. Comprehensive composition analyses for L-rich and S-rich LCDs in yeast.** Complete composition analyses were performed as described for Fig 7.

Fig S15

**Fig S15. Removal of intraclass high-homology proteins does not dramatically influence the majority of LCD functional associations.** (A) Percent sequence identity based on multiple sequence alignment (performed using the Clustal Omega server; https://www.ebi.ac.uk/Tools/msa/clustalo/) of all proteins in each LCD class. (B) For each secondary amino acid LCD subclass, functional associations were evaluated before and after removal of proteins with >80% sequence identity for any other protein in that LCD subclass. The stacked barchart indicates the proportion of originally-observed GO terms that were still detected (blue) or not detected (grey) after high-homology proteins were removed. (C) Identical to the analyses in panel B, but for multi-LCD subclasses.

Supplementary Table Legends

**Table S1. Total LCD content by LCD class for all reference proteomes.** For each organism, the total percentage of the proteome classified as LCD for each LCD class was calculated as the percentage of amino acids found within LCD regions. UniProt taxon IDs were mapped to the scientific name and common name (when available) for each organism using taxonomy data downloaded from (https://www.uniprot.org/taxonomy/).

**Table S2. Cross-domain comparison of LCD content distributions for each LCD class.** For each domain of life, organisms were stratified into one of seven categorical bins based on the percentage of the proteome classified as LCD for each LCD class [None (0%), Extremely Low (0-0.1%), Very Low (0.1-0.5%), Low (0.5-2%), Medium (2-5%), High (5-10%), Very High (10-15%), or Extremely High (>15%)]. The proportion of total proteomes for each domain of life was then calculated for each bin (see also Fig 4).

**Table S3. Comprehensive listing and composition analyses of all LCDs identified across 7 model eukaryotic organisms.** LCDs were identified in the 7 model eukaryotic organisms by running LCD-Composer with default parameters for each of the 20 canonical amino acids, then collected into a single file.

**Table S4. Combined GO term results for LCD-associated functions across 7 model eukaryotic organisms.** GO term analyses were performed separately for each LCD class and organism. For each LCD class, the number of times each significantly enriched GO term occurred across the 7 model eukaryotic organisms is indicated, as well as the corresponding organism(s).

**Table S5. Statistically significant GO term results for all LCD classes across model organisms.** GO term analyses were performed separately for each LCD class and organism. All statistically significant results (Šidák-corrected *P* < 0.05) were collated into a single list, with each organism and LCD class indicated.

**Table S6. Statistical comparison of GO term overlap for homotypic vs. heterotypic LCD class comparisons.** For all pairwise organism permutations, the proportion of GO terms significantly enriched for a given LCD class that overlap with each LCD class of the secondary organism was calculated. This process was repeated for all LCD classes for the primary organism, resulting in a 20x20 matrix of overlap proportion values for each pairwise organism permutation (see Fig S7). Homotypic LCD class comparisons (e.g. A-rich LCDs from organism1 vs. A-rich LCDs from organism2) are represented by on-diagonal values, whereas heterotypic LCD class comparisons (A-rich LCDs from organism1 vs. E-rich LCDs from organism2) are represented by off-diagonal values, expressed as a concatenated list of comma-separated values taken from left-to-right and top-to-bottom from each matrix. All comparisons for which the enriched GO term sample size was zero for either of the LCD classes from the two organisms (values in the table beginning with an asterisk) were excluded from statistical analyses. For each pairwise organism permutation, the distribution of on-diagonal values was statistically compared to the distribution of off-diagonal values using a two-sided Mann-Whitney *U* test.

**Table S7. Statistical evaluation of the the number of times each GO term was enriched across 7 eukaryotic model organisms.** Observed cross-organism GO term counts for LCD classes (Table S4) were compared to cross-organism GO term counts derived from random sampling, based on random sampling of GO terms, using a two-tailed Fisher’s exact test (see Fig S11 legend for full description). Odds ratios listed as “inf” represent categories with a non-zero observed cross-organism count but zero occurrences from random sampling (resulting in a zero in the odds ratio denominator). Odds ratios listed as “nan” represents cases in which both the observed and sampling-derived counts are zero.

**Table S8. Statistical estimation of the degree of enrichment or depletion of secondary amino acid subclasses for each primary LCD class.** Within each primary LCD class, the frequency of LCDs assigned to each LCD subclass was compared to corresponding scaled whole-proteome frequencies using a two-tailed Fisher’s exact test (see Material and Methods for full description). Subclasses for which the scaled whole-proteome frequency was <1 were excluded from the calculations (indicated with “nan” for *P*-value, corrected *P*-value, odds ratio, and lnOR). Subclasses with a scaled whole-proteome frequency >1 and no observed LCDs were assigned an imputed value of 1 (indicated in parentheses) to serve as a conservatively biased estimate.

**Table S9. GO term results from subclassification of LCDs based on a secondary amino acid for all model organisms.** All LCDs composed of ≥40% composition of a primary search amino acid and ≥20% of a second amino acid were identified in each model organism proteome using LCD-Composer’s multifaceted composition criteria (with otherwise default parameters). GO term analyses were performed for each LCD “subclass” (e.g. A-rich LCDs with a secondary bias for G residues). GO terms associated with the original primary LCD classes (Table S5) for the corresponding organism could be either “Lost” (were not significantly enriched for any LCD subclass), “Retained” (significantly enriched for at least one LCD subclass), or “New” (significantly enriched for at least one LCD subclass but not enriched for the original primary LCD class).

**Table S10. Estimation of the relative degree of GO term enrichment upon LCD subclassification by secondary amino acid.** For all “retained” and “new” GO terms observed upon subclassification of LCDs based on a secondary amino acid (Fig 9 and Table S9), the degree of GO term enrichment was calculated as the difference in the natural log of the odds ratios. Additionally, 95% confidence intervals were calculated for 1) the odds ratio of the proteins in the primary LCD set relative to the whole proteome, and 2) the odds ratio of the proteins in the subclassification LCD set relative to the whole proteome, then tested for overlap.

**Table S11. GO term results from subclassification of multi-LCD proteins based on co-occurring LCDs for all model organisms.** Proteins with multiple non-overlapping LCDs of distinct classes were parsed into each pairwise amino acid subclass based on the predominant residue in each non-overlapping LCD. GO terms associated with the original primary LCD classes (Table S5) for the corresponding organism could be either “Lost” (were not significantly enriched for any LCD subclass), “Retained” (significantly enriched for at least one LCD subclass), or “New” (significantly enriched for at least one LCD subclass but not enriched for the original primary LCD class).

**Table S12. Estimation of the relative degree of GO term enrichment for multi-LCD proteins.** Analyses were identical to those described for Table S10 but for multi-LCD subclassification (corresponding to Fig 10 and Table S11).

References

1. Cascarina,S.M. and Ross,E.D. (2018) Proteome-scale relationships between local amino acid composition and protein fates and functions. *PLOS Comput. Biol.*, **14**, e1006256.

2. Cascarina,S.M., Elder,M.R. and Ross,E.D. (2020) Atypical structural tendencies among low-complexity domains in the protein data bank proteome. *PLoS Comput. Biol.*, **16**.

3. Van Roey,K., Uyar,B., Weatheritt,R.J., Dinkel,H., Seiler,M., Budd,A., Gibson,T.J. and Davey,N.E. (2014) Short linear motifs: Ubiquitous and functionally diverse protein interaction modules directing cell regulation. *Chem. Rev.*, **114**, 6733–6778.
